# Using Integrated Network Pharmacology and Metabolomics to Reveal the Mechanisms of the Combined Intervention of Ligustrazine and Sinomenine in CCI-Induced Neuropathic Pain Rats

**DOI:** 10.3390/ijms26062604

**Published:** 2025-03-13

**Authors:** Zhaoyue Yuan, Xiaoliang Zhao, Yan Zhang, Yue Jiao, Yang Liu, Chang Gao, Jidan Zhang, Yanyan Ma, Zhiguo Wang, Tao Li

**Affiliations:** 1 Experimental Research Center, China Academy of Chinese Medical Sciences, Beijing 100700, China; zyueyuan1118@163.com (Z.Y.); zhaoxiaoliang@merc.ac.cn (X.Z.);; 2 Institute of Chinese Materia Medica, China Academy of Chinese Medical Sciences, Beijing 100700, China; 178062972336@163.com

**Keywords:** neuropathic pain, ligustrazine, sinomenine, tyrosine metabolism, phenylalanine metabolism

## Abstract

Neuropathic pain (NP) is a type of chronic pain resulting from injury or dysfunction of the nerves or spinal cord. Previous studies have shown that the combination of ligustrazine (LGZ) and sinomenine (SIN) exerts a synergistic antinociceptive effect in peripheral and central NP models. On this basis, a comprehensive analgesic evaluation was performed in a chronic constriction injury (CCI)-induced NP model in rats. Sciatic nerve histopathological changes were observed, and 22 cytokines and chemokines levels were analyzed. We also combined network pharmacology and metabolomics to explore their molecular mechanisms. Results showed that the combination of LGZ and SIN significantly alleviated the pain-like behaviors in CCI rats in a time- and dose-dependent manner, demonstrating superior therapeutic effects compared to LGZ or SIN alone. It also improved pathological damage to sciatic nerves and regulated inflammatory cytokine levels. Network pharmacology identified shared and distinct pain-related targets for LGZ and SIN, while metabolomics revealed 54 differential metabolites in plasma, and 17 differential metabolites in CSF were associated with the combined intervention of LGZ and SIN. Finally, through an integrated analysis of the core targets and differential metabolites, tyrosine metabolism, phenylalanine metabolism, and arginine and proline metabolism were identified as potential key metabolic pathways underlying the therapeutic effects of LGZ and SIN in CCI treatment. In conclusion, our study provides evidence to support the clinical application of LGZ and SIN in the treatment of NP.

## 1. Introduction

Neuropathic pain (NP) is a chronic condition that is characterized by spontaneous and evoked pain, including cold and mechanical allodynia. NP arises from injury or malfunction of the nerves or the spinal cord, and is prevalent in clinical practice. It often occurs secondary to conditions such as trauma, stroke, infection, diabetes, multiple sclerosis, and cancer [1,2]. Globally, the incidence of NP accounts for 6.9% to 10% of the total population [3]. NP leads to significant reductions in both quality of life and behavioral function, placing a heavy physical and psychological burden on patients [4]. The pathophysiology of NP is complex and multifactorial. Despite the availability of various interventions based on different mechanisms, the results of NP treatment are still unsatisfactory [5]. Commonly used medications for NP, such as non-steroidal anti-inflammatory drugs and opioids, often cause adverse effects, including dizziness, drowsiness, arrhythmias, and issues related to tolerance when used alone [6]. Consequently, the development of combination therapies targeting multiple analgesic mechanisms has become a promising strategy in the treatment of NP. Indeed, clinical approaches to chronic pain often evolve from initial monotherapy to combination therapy as a more effective means of addressing the complex nature of NP [7].

Traditional Chinese medicine (TCM) boasts an extensive history in the treatment of NP, with many herbal medicines and their active compounds demonstrating analgesic effects [8]. These therapies represent a rich source for the screening of potential combination drugs for NP treatment. As reported, ligustrazine (LGZ) has exhibited therapeutic effects, such as alleviating migraine [9] and spinal cord injury-induced NP [10], in various pain animal models. Sinomenine (SIN) has demonstrated analgesic effects on inflammatory pain [11], spared nerve injury-induced NP [12], and diabetic peripheral NP [13], exerting both anti-inflammatory and analgesic mechanisms. Our previous studies have shown that the combination of LGZ and SIN effectively alleviates pain in NP models, including sciatic nerve injury, trigeminal neuralgia, and spinal cord injury [14]. Moreover, the combination of LGZ and SIN exhibits a synergistic analgesic effect, allowing for a reduction in the individual dosages of each compound. Both LGZ and SIN have been reported to have numerous potential targets and pathways, but the specific mechanisms underlying their analgesic effects in NP remain unclear.

Network pharmacology is used to analyze the relationships between drugs, targets, and diseases through network-based approaches, making it particularly well-suited for elucidating core targets and pathways in treating complex diseases via TCM, which has “multi-target” and “multi-pathway” characteristics [15]. Through offering a systems-level perspective, network pharmacology uncovers the potential molecular mechanisms of TCM, shedding light on its holistic therapeutic effects [16,17]. Metabolomics, an essential component of systems biology, utilizes high-throughput and highly sensitive instruments to conduct comprehensive profiling of the endogenous components within biological samples. Through integrating multivariate statistical methods, metabolomics can reveal changes in endogenous metabolites under various physiological, pathological, or toxicological conditions [18], enabling the identification of the key metabolic pathways within the organism [19]. Therefore, the integration of metabolomics with network pharmacology can provide a more comprehensive understanding of the metabolic pathways and network regulatory mechanisms of the combined therapy in treating complex diseases.

In this study, we employed network pharmacology to predict the potential targets of LGZ and SIN in treating pain-related diseases, systematically analyzing both their shared and distinct targets. Subsequently, the analgesic effects of LGZ, SIN, and their combination at different time points and dosages were evaluated in a chronic constriction injury (CCI)-induced NP model in rats. Furthermore, the key metabolites in the plasma and cerebrospinal fluid (CSF) were identified and quantified using LC-MS metabolomics technology. Finally, a joint analysis of the potential targets and key metabolites was performed to determine the critical metabolic pathways through which LGZ and SIN exert their therapeutic effects in NP. This study aimed to elucidate the molecular mechanisms underlying the combined use of LGZ and SIN in treating NP. Additionally, this research aimed to offer new experimental evidence to support the clinical combined application of LGZ and SIN in treating NP.

## 2. Results

### 2.1. The Combination of LGZ and SIN Pharmacological Effects on CCI Rats

#### 2.1.1. General Animal Condition

**Figure 1:**
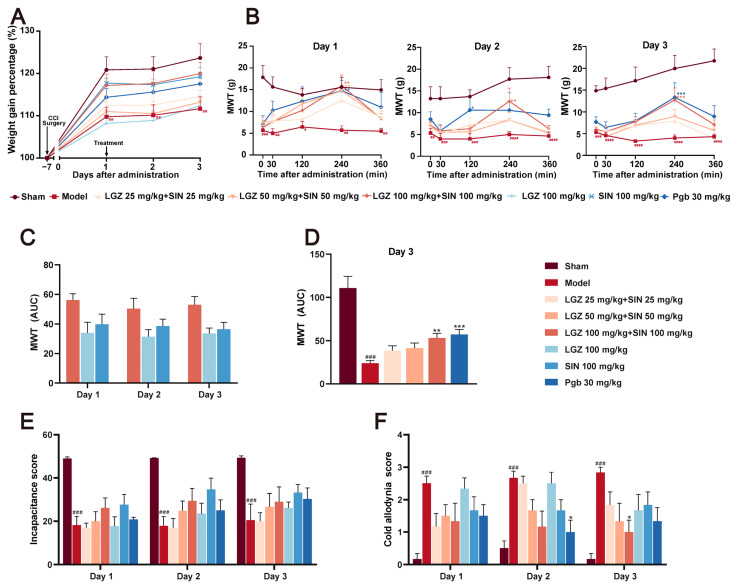
The analgesic effects of different doses of SIN + LGZ (LGZ 25 mg⋅kg^−1^⋅d^−1^ + SIN 25 mg⋅kg^−1^⋅d^−1^; LGZ 50 mg⋅kg^−1^⋅d^−1^ + SIN 50 mg⋅kg^−1^⋅d^−1^; and LGZ 100 mg⋅kg^−1^⋅d^−1^ + SIN 100 mg⋅kg^−1^⋅d^−1^), SIN (100 mg⋅kg^−1^⋅d^−1^), and LGZ (100 mg⋅kg^−1^⋅d^−1^) in CCI rats following intragastric administration on days 1, 2, and 3. (**A**) Body weight gain percentages. (**B**) Mechanical withdrawal threshold (MWT) values after administration over 3 days of treatment. (**C**) The AUC of the MWT values on the third day of treatment. (**D**) The AUC of the MWT values per day over 3 days of treatment. (**E**) The cold allodynia score over 3 days of treatment. (**F**) The incapacitance score over 3 days of treatment. ^#^ *p* < 0.05, ^##^ *p* < 0.01, ^###^ *p* < 0.001 and ^####^ *p* < 0.001 (compared to the sham group). * *p* < 0.05, ** *p* < 0.01, and *** *p* < 0.001 (compared to the model group).

Following CCI surgery and treatment, all of the rat groups exhibited an upward trend in body weight (Figure 1A). After administration, the model group had a slower weight gain than the sham group. Conversely, all treated groups exhibited greater body weight gain compared to the model group, with the LGZ+SIN high-dose group showing the most significant increase. These findings suggested that the combined treatment of LGZ and SIN has a restorative effect on the overall status of CCI rats. No significant toxic effects were observed in any of the groups throughout the experiment.

#### 2.1.2. The Combination of LGZ and SIN Improve the Pain-Related Behaviors in CCI Rats

The mechanical withdrawal threshold (MWT) results indicated that, as early as 0.5 h post-administration, the LGZ+SIN, LGZ, and SIN groups significantly increased MWT, peaking at 4 h, with statistically significant differences. The pain-relieving trend remained consistent across all treatment groups on days 1, 2, and 3 post-treatment (Figure 1B). To further quantify the effects, we calculated the area under the curve (AUC) of the MWT values at 0, 0.5, 2, 4, and 6 h post-treatment. The MWT values were measured on the first, second, and third days after administration, and they were used to calculate the AUC for the LGZ+SIN high-dose, LGZ, and SIN groups. The results showed that the AUC for the MWT values in the LGZ+SIN groups was consistently superior to that of either the LGZ or SIN groups alone on all days of administration (Figure 1C). Furthermore, the LGZ+SIN high-dose group increased the AUC for MWT more than LGZ+SIN medium- or low-dose group on the third day of treatment (Figure 1D). These results suggest that the combined use of LGZ and SIN effectively alleviated mechanical pain in CCI rats, delivering a superior effect compared to the individual use of LGZ or SIN.

The cold allodynia test results (Figure 1E) revealed that, during the administration period, the cold pain scores of the model group were significantly higher than those of the Sham group (*p* < 0.001), indicating hypersensitivity to cold pain in the CCI rats. Over the 3-day treatment period, the cold pain sensitivity scores in the LGZ+SIN and SIN groups showed a decreasing trend compared to the model group. On the third day of administration, the cold pain score in the LGZ+SIN high-dose group was significantly reduced (*p* < 0.05). These results suggest that the combined use of LGZ and SIN has a significant effect in alleviating the cold pain sensitivity in CCI rats. As shown in Supplementary Figure S1, the cold allodynia score in the LGZ+SIN groups decreased significantly at 4 h post-administration, especially in the LGZ+SIN high-dose group. The results indicate that the combination of LGZ and SIN has the best effect after 4 h of administration.

The results of the incapacitance test (Figure 1F) showed that, compared to the sham group, the model group had a significantly reduced incapacitance score (*p* < 0.01), indicating the presence of spontaneous pain in the CCI rats. Compared to the model group, the incapacitance score in the LGZ+SIN, LGZ, and SIN groups showed an increasing trend following treatment, with more pronounced improvements on the second and third days of administration. These results suggested that the combined use of LGZ and SIN might alleviate spontaneous pain in CCI rats.

#### 2.1.3. The Combination of LGZ and SIN Alleviates the Pathological Structure of the Sciatic Nerves

**Figure 2:**
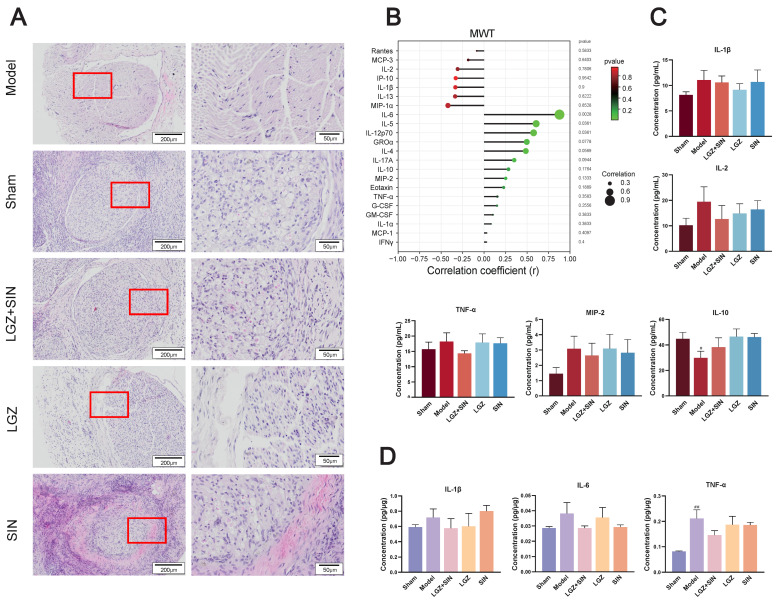
LGZ+SIN-attenuated, CCI-induced sciatic nerve injury. (**A**) Representative images of HE staining of sciatic nerves. The red frame in the left images are enlarged and displayed on the right side. (**B**) Correlation coefficient chart between MWT values and 22 cytokines and chemokines. The size of the dots represents the strength of the association between the MWT values and 22 cytokines and chemokines; a larger dot indicates a stronger correlation. The color represents the *p*-value, with redder colors indicating larger *p*-values. (**C**) Inflammatory factor changes in a plasma sample. (**D**) Inflammatory factor changes in a sciatic nerve sample. ^#^ *p* < 0.05, and ^##^ *p* < 0.01 (compared to the sham group).

H&E staining of the sciatic nerves revealed that the sciatic nerve fibers in the sham group rats had a regular, evenly distributed structure, with tightly arranged, intact myelin sheaths. In contrast, the model group rats showed a loose, sparse arrangement of sciatic nerve fibers, with deformed myelin sheaths and a significant infiltration of inflammatory cells. The sciatic nerve structure in the LGZ group was closer to normal, with a more orderly arrangement. Although demyelination was present, it was less severe than in the model group, indicating a degree of pathological recovery. Both the LGZ+SIN and SIN groups exhibited varying degrees of disorganized nerve fiber arrangement, demyelination, and inflammatory cell infiltration, but the extent of these changes was milder than in the model group (Figure 2A). These results suggest that the combined use of LGZ and SIN, LGZ, and SIN has a beneficial effect on the pathological repair of the sciatic nerves in CCI rats.

#### 2.1.4. The Combination of LGZ+SIN Alter the Plasma and Sciatic Nerve Inflammatory Cytokine and Chemokine Levels in CCI Rats

As shown in Figure 2B, we performed a correlation analysis of 22 cytokines and chemokines with MWT. The results indicate that MIP-1α, IL-13, IL-1β, IP-10, IL-2, MCP-3, rates are negatively correlated with MWT, while IL-6, IL-5, IL-12p70, GROα, IL-4, IL-17A, IL-10, MIP-2, and others are positively correlated with MWT.

The concentrations of TNF-α, IL-1β, IL-2, and MIP-2 in the plasma of each group (Figure 2C) showed that, compared to the sham group, the model group had higher levels. After three days of treatment, the LGZ+SIN group exhibited a decreased level compared to the model group. Additionally, the concentrations of IL-10 in the model group decreased compared with the sham group. After treatment, the LGZ+SIN group decreased. These results suggest that the combined use of LGZ and SIN has a beneficial effect in alleviating plasma inflammation in CCI rats.

The concentrations of IL-1β, IL-6, and TNF-α in the sciatic nerves of each group (Figure 2D) showed that, compared to the sham group, the model group had higher levels of IL-1β, IL-6, and TNF-α. After three days of treatment, the LGZ+SIN group exhibited a decreased level in the sciatic nerves compared to the model group. These results suggest that the combined use of LGZ and SIN has a beneficial effect in alleviating sciatic nerve inflammation in CCI rats.

### 2.2. Results of Network Pharmacology Analysis

#### 2.2.1. Potential Targets of LGZ and SIN in Pain Treatment

A compound target database search and screening identified 29 targets for LGZ and 100 targets for SIN. The GeneCards database revealed a total of 12,990 pain-related targets, with relevance scores ranging from a maximum of 100.27 to a minimum of 0.08. Targets with scores above the median were screened, resulting in 3303 pain-related targets. After merging the results from multiple databases and removing duplicates, a final set of 3303 disease-related targets was obtained.

**Figure 3:**
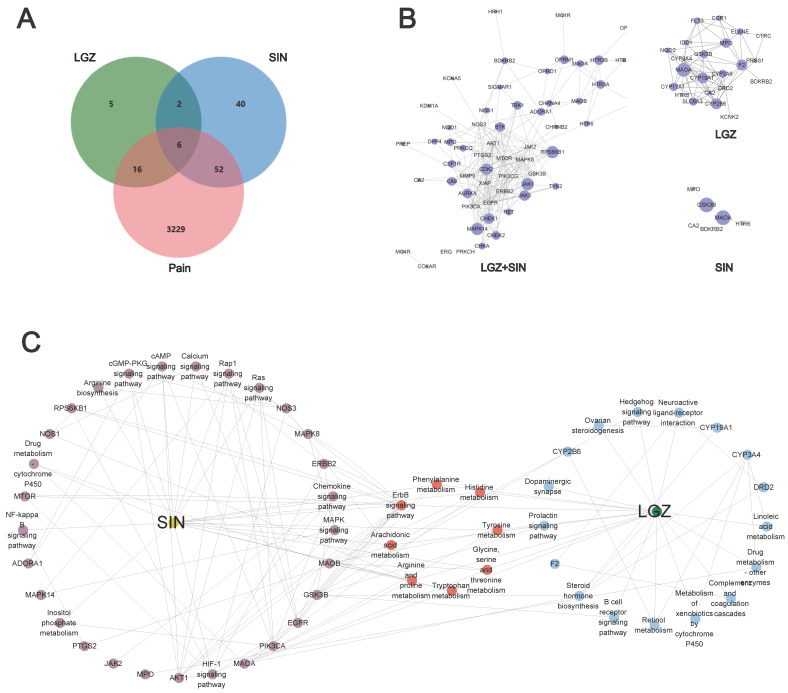
Exploring the effects of LGZ and SIN on pain based on network pharmacology. (**A**) Venn diagram. (**B**) Protein–protein network diagram. (**C**) Target–pathway networks of LGZ and SIN through KEGG enrichment analysis of the targets.

The target information of the compounds was intersected with disease-related target genes on the GeneCards database, and a Venn diagram was constructed (Figure 3A). This intersection revealed 22 pain-related targets for LGZ, 58 pain-related targets for SIN, and 8 common targets shared by both compounds. Among these, 16 specific targets were identified for LGZ, and 52 unique targets were identified for SIN. These intersected genes were then entered into the STRING 11.0 platform to construct interaction networks based on protein–protein interactions (Figure 3B).

This analysis revealed that LGZ and SIN exerted their analgesic effects through six key targets: CA2, MPO, HTR6, MAOA, GSK3B, and BDKRB2. Additionally, both compounds exhibited analgesic effects through their respective specific targets.

#### 2.2.2. Pain-Relieving Pathways and Biological Function Differences Between LGZ and SIN

LGZ and SIN exhibited certain differences in their pain-relieving pathways and biological functions. The core targets of both compounds were analyzed using Metascape, and KEGG and GO functional enrichment analyses were performed (*p* < 0.01). The top 30 GO terms were selected for the bar chart, and the top 20 KEGG pathways were visualized in a bubble chart (Supplementary Figure S2A,B).

According to the KEGG analysis, the primary targets of LGZ are related to the nervous system (dopaminergic), signaling pathways (synapse neuroactive ligand–receptor interaction, hedgehog signaling pathway, and ErbB signaling pathway), the immune system (B cell receptor signaling pathway), and the endocrine system (prolactin signaling pathway). It also modulated the amino acid metabolism pathways such as tyrosine metabolism and tryptophan metabolism to exert its analgesic effect. Additionally, SIN exerted its analgesic effects by modulating signaling pathways (PI3K-Akt signaling pathway, TNF signaling pathway, sphingolipid signaling pathway, and ErbB signaling pathway), the nervous system (dopaminergic), the immune system (IL-17 signaling pathway and T cell receptor signaling pathway), and the endocrine system (prolactin signaling pathway, insulin signaling pathway, growth hormone synthesis, secretion, and action).

Venn diagram analysis revealed that LGZ and SIN share six common analgesic targets. By integrating these targets with the KEGG analysis, their intersecting genes primarily exerted analgesic effects by influencing the nervous system (serotonergic synapse and dopaminergic synapse), signaling pathways (calcium signaling pathway, neuroactive ligand–receptor interaction, and hedgehog signaling pathway), and the endocrine system (prolactin signaling pathway). These pathways, along with energy metabolism and amino acid metabolism pathways, were key to their analgesic effects.

To further clarify the combination pathways of LGZ and SIN for treating NP, all of the targets were mapped onto KEGG pathways to identify the pathways with *p* < 0.05. The pathways were selected, resulting in a target–pathway network (Figure 3C). The results showed that LGZ and SIN shared eight signaling pathways. These results demonstrated that the combined treatment of LGZ and SIN for NP was a form of combination therapy.

In summary, LGZ and SIN share six common pain-relieving target genes: CA2, HTR6, MAOA, GSK3B, BDKRB2, and MPO. An analysis of the core targets revealed that both compounds primarily affect the nervous system, immune system, and signaling pathways to exert their analgesic effects. However, a closer examination of the intersecting genes reveals that HTR6 and BDKRB2 are highly expressed in signaling pathways, while CA2 shows strong expression in the neuroactive ligand–receptor interaction pathway. Furthermore, the biological processes of these common genes suggest that they mainly influence neurotransmitter breakdown and ion transport processes. This indicates that the combined use of LGZ and SIN might, to some extent, enhance the neuroprotective and signaling function of the system.

### 2.3. Metabolomic Analysis

A widely targeted metabolomics approach was employed to investigate the therapeutic mechanism of the combined use of LGZ and SIN, as well as their individual applications on CCI rats. To gain more detailed insights, plasma and CSF samples were analyzed separately, in both positive and negative ion modes. The results from the positive and negative ion detection were subsequently merged and further analyzed to explore the underlying therapeutic mechanisms of treatment.

#### 2.3.1. Differential Metabolites Analysis

Principal component analysis (PCA) was performed on the data from each group. The PCA score plot revealed that the QC samples exhibited excellent stability, indicating that the analytical system had strong reliability, and that the experimental data were of high quality. This suggests that the data met the criteria for metabolomics analysis. The details can be found in Supplementary Figure S3.

**Figure 4:**
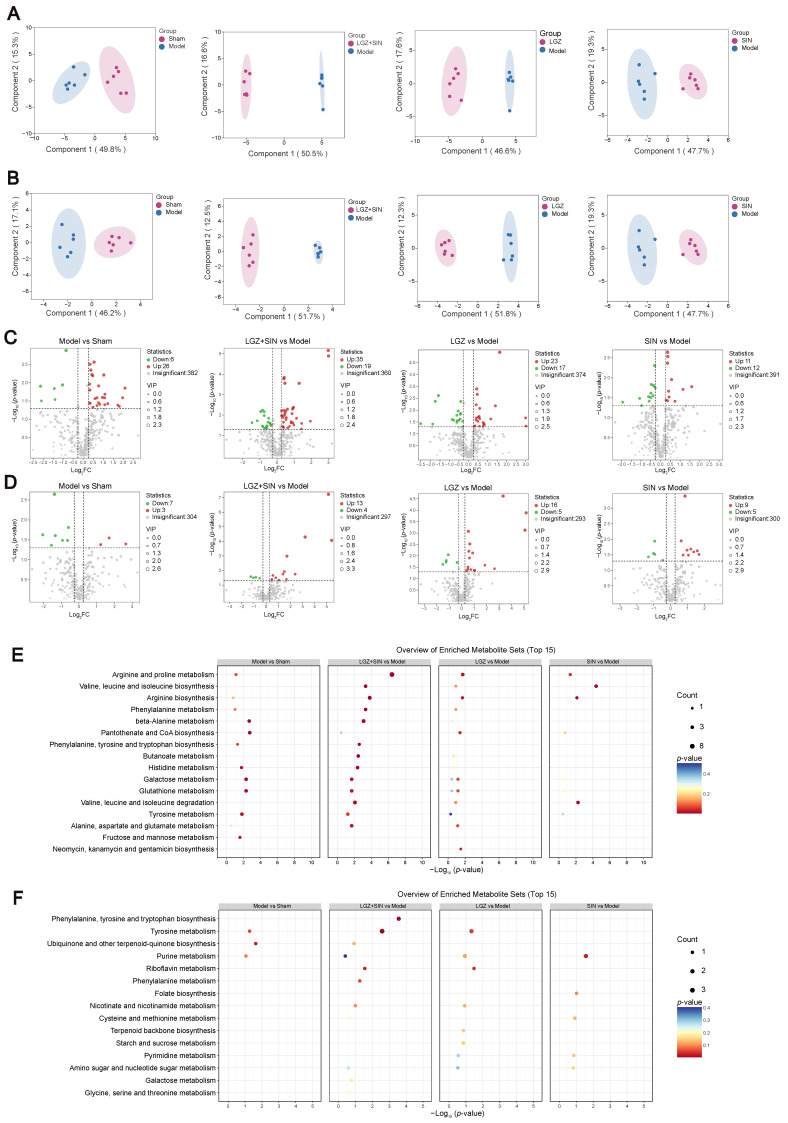
The metabolite changes in CCI rats following LGZ+SIN intervention. (**A**) Results of the OPLS-DA scores’ scatter plot of the plasma samples. (**B**) Results of the OPLS-DA scores’ scatter plot of the CSF samples. (**C**) Volcano plot of the metabolites in the plasma samples. (**D**) Volcano plot of the metabolites in the CSF samples. (**E**) Top 15 enriched pathways for differential metabolites upon plasma samples. (**F**) Top 15 enriched pathways for differential metabolites upon CSF samples. Data were analyzed by MetaboAnalyst 6.0 using KEGG metabolite sets library.

Orthogonal partial least squares discriminant analysis (OPLS-DA) was used to further identify the potential biomarkers between the groups in the plasma and CSF samples, respectively (Figure 4A,B). The OPLS-DA score plots clearly distinguished between the groups, indicating significant differences in metabolic profiles and suggesting that endogenous metabolites underwent noticeable changes among the groups.

To assess the robustness and validity of the OPLS-DA models, a random permutation test (*n* = 200) was performed (Supplementary Figure S4). The results indicate that none of the models were overfitted, confirming that the modeling outcomes were reliable. A model is considered valid when both the R^2^Y and Q^2^ values are greater than 0.5, which demonstrates good predictive power and confirms that the data are reliable.

To further validate the results, a *t*-test was conducted to examine whether significant differences existed between the groups. Differential metabolites between the sham and model groups, as well as between the treatment groups and the model group, were identified. As shown in Figure 4C,D, the metabolites that increased significantly (*p* < 0.05) with a FC > 1.2 are marked in red, and metabolites that decreased significantly (*p* < 0.05) and exhibited a FC < 0.83 are marked in green.

In the plasma samples, a total of 32 differential metabolites were identified between the model and sham groups, with 26 metabolites upregulated and 6 downregulated. Compared to the model group, 54 differential metabolites were identified in the LGZ+SIN group, including 35 upregulated and 19 downregulated. In the LGZ group, 40 differential metabolites were detected compared to the model group, with 23 upregulated and 17 downregulated. In the SIN group, 23 differential metabolites were identified compared to the model group, with 11 upregulated and 12 downregulated. From the perspective of the number of differential metabolites, the LGZ+SIN group exhibited a total of 54 differential metabolites, which was 14 more than those in the LGZ group and 31 more than those in the SIN group. In the CSF samples, 10 differential metabolites were identified between the model and sham groups, with 3 metabolites upregulated and 7 downregulated. In the LGZ+SIN group, compared to the model group, 17 differential metabolites were identified, with 13 upregulated and 4 downregulated. In the LGZ group compared to the model group, 21 differential metabolites were identified, with 16 upregulated and 5 downregulated. In the SIN group, 14 differential metabolites were identified, with 9 upregulated and 5 downregulated. From the perspective of the number of differential metabolites, the LGZ+SIN group had a greater effect on the changes in the metabolic profile in CSF.

We performed correlation analysis between differential metabolites and MWT in plasma and CSF of the LGZ+SIN group, respectively (Supplementary Figure S5A,B). As shown in the figure, the differential metabolites in plasma, including phenylacetaldehyde, L-Ornithine, and 5-hydroxytryptophan, were significantly correlated with MWT (Mantel’s r ≥ 0.4, *p* < 0.01). Similarly, the differential metabolites in CSF, including N, N-dimethyl-1,4-phenylenediamine, L-gulose, dethiobiotin, and phenylpyruvate, also showed significant correlations with MWT (Mantel’s r ≥ 0.4, *p* < 0.01). Additionally, we performed correlation analysis between the differential metabolites and 22 cytokines and chemokines in the plasma and CSF of the LGZ+SIN group (Supplementary Figure S5C,D). Differential metabolites in plasma, including 1-aminocyclopropanecarboxylate, succinate semialdehyde, pentanoate and cortexolone, were correlated with cytokines and chemokines (Mantel’s r ≥ 0.4, *p* < 0.05). And riboflavin in CSF was correlated with cytokines and chemokines (Mantel’s r ≥ 0.4, *p* < 0.01). These results suggest that changes in differential metabolites are closely associated with pain-like behavior and inflammatory factors in plasma.

#### 2.3.2. Metabolomic Pathway Analysis

To identify the metabolic pathways most relevant to the pain-relieving effect of the combined use of LGZ and SIN in CCI rats, MetaboAnalyst 6.0 software was used to analyze the metabolic pathways. Pathways with an impact value (impact > 0) were selected as the screening criterion.

In the plasma samples, the model group primarily interfered with 28 metabolic pathways, including tyrosine metabolism; phenylalanine, tyrosine and tryptophan biosynthesis; and beta-alanine metabolism, which contribute to the development of neuropathic pain and related symptoms. The LGZ+SIN group primarily modulated 29 metabolic pathways, including arginine and proline metabolism, phenylalanine metabolism, phenylalanine, tyrosine and tryptophan biosynthesis, arginine biosynthesis, alanine, aspartate and glutamate metabolism, beta-alanine metabolism, and tyrosine metabolism. The LGZ group primarily modulated 28 pathways, including arginine biosynthesis, arginine and proline metabolism, and phenylalanine metabolism. The SIN group primarily regulated 14 pathways, including cysteine and methionine metabolism, phenylalanine, tyrosine and tryptophan biosynthesis, phenylalanine metabolism, and arginine biosynthesis. From the perspective of the number of metabolic pathways regulated, the LGZ+SIN group influenced one more pathway than LGZ and fourteen more than SIN. Moreover, LGZ+SIN regulated several metabolic pathways that involved four or more differential metabolites, facilitating the identification of key metabolic pathways (Figure 4E).

In the CSF samples, the model group primarily modified three pathways, including tyrosine metabolism and purine metabolism, contributing to the occurrence of neuropathic pain-related symptoms. The LGZ+SIN group primarily modulated 12 pathways, including tyrosine metabolism, riboflavin metabolism, and phenylalanine metabolism. The LGZ group regulated 11 pathways, including tyrosine metabolism, riboflavin metabolism, and tryptophan metabolism. The SIN group primarily affected five pathways, including purine metabolism (Figure 4F).

**Figure 5:**
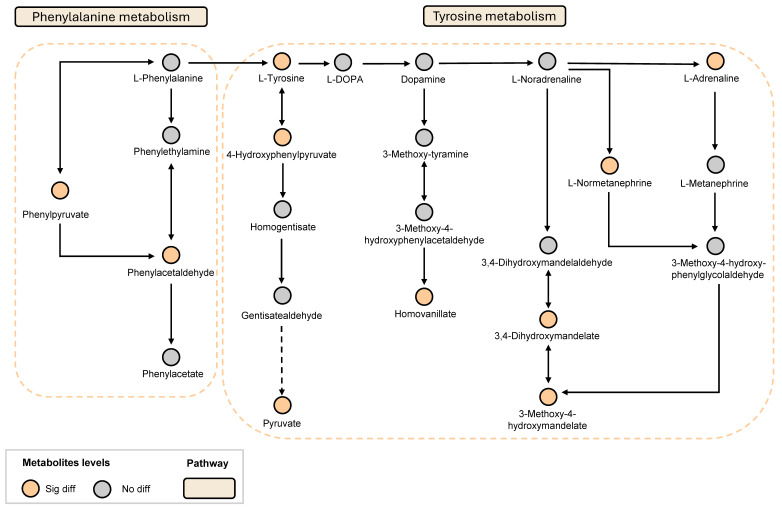
LGZ and SIN intervened in the key metabolic pathways of the CCI rats. Orange circles indicate metabolites that have significant difference. Gray circles indicate metabolites that are not different.

Based on these results and in conjunction with the KEGG database, the key metabolic pathways involved in LGZ and SIN intervention in NP include the tyrosine metabolism pathway and the phenylalanine metabolism pathway (Figure 5).

#### 2.3.3. The Combination of LGZ and SIN Altered the Metabolic Profile of CCI Rats

**Figure 6:**
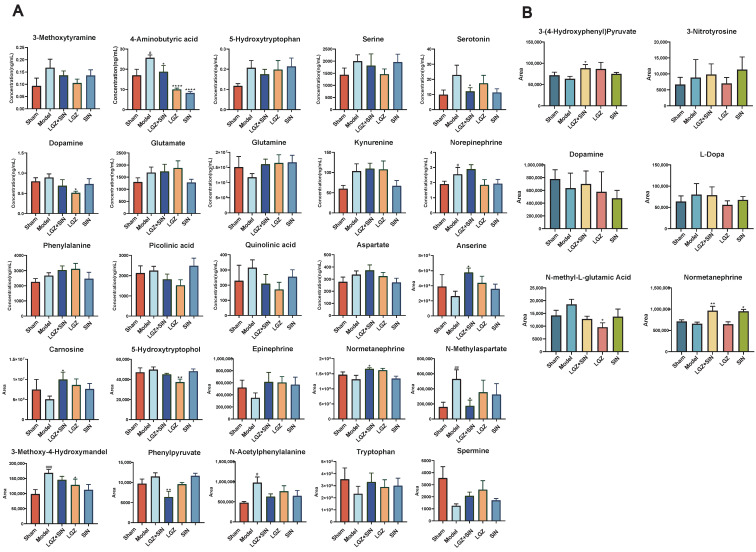
LGZ+SIN altered the metabolites of the biological samples in the CCI rats. (**A**) The differential metabolites in plasma. (**B**) The differential metabolites in CSF. ^#^ *p* < 0.05, ^##^ *p* < 0.01, and ^###^ *p* < 0.001 (compared to the sham group). * *p* < 0.05, ** *p* < 0.01 and **** *p* < 0.0001 (compared to the model group).

In the plasma samples, LGZ+SIN significantly increased the levels of 5-hydroxytryptophan, normetanephrine, anserine, and carnosine, while significantly reducing the levels of phenylpyruvate and N-methylaspartate. Additionally, LGZ+SIN significantly increased the levels of epinephrine, tryptophan, and spermine, while decreasing the levels of 3-methoxy-4-hydroxymandelate and N-acetylphenylalanine. LGZ significantly increased the levels of 5-hydroxytryptophan and notably decreased the levels of 5-hydroxytryptophol and 3-methoxy-4-hydroxymandelate. LGZ also increased the levels of epinephrine, normetanephrine, tryptophan, spermine, anserine, and carnosine, while decreasing the levels of phenylpyruvate, N-acetylphenylalanine, and N-methylaspartate. SIN significantly elevated the levels of 5-hydroxytryptophan, epinephrine, tryptophan, spermine, anserine, and carnosine, while notably decreasing the levels of 3-methoxy-4-hydroxymandelate, N-acetylphenylalanine, and N-methylaspartate. In the targeted analysis of neurotransmitter-related metabolites in plasma samples, LGZ+SIN significantly decreased the levels of 4-aminobutyric acid and serotonin, while notably improving the levels of 3-methoxytyramine, 5-hydroxytryptophan, choline, dopamine, glutamine, picolinic acid, and quinolinic acid. LGZ and SIN both significantly decreased the levels of 4-aminobutyric acid. LGZ also significantly reduced the level of dopamine, while improving the levels of 3-methoxytyramine, aspartate, choline, glutamine, norepinephrine, picolinic acid, quinolinic acid, serine, and serotonin. SIN notably improved the levels of 3-methoxytyramine, aspartate, dopamine, glutamate, glutamine, kynurenine, norepinephrine, phenylalanine, quinolinic acid, and serotonin (Figure 6A).

In the CSF samples, the combination of LGZ and SIN significantly rebalanced the levels of 3-(4-hydroxyphenyl) pyruvate and normetanephrine, and notably adjusted the levels of dopamine and L-dopa, while reducing the level of N-methyl-L-glutamic acid. LGZ significantly reduced the level of N-methyl-L-glutamic acid, while also rebalancing 3-(4-hydroxyphenyl) pyruvate and reducing 3-nitrotyrosine, dopamine, and L-dopa levels. SIN significantly rebalanced normetanephrine levels, and notably adjusted the levels of L-dopa and N-methyl-L-glutamic acid (Figure 6B).

### 2.4. Joint Pathway Analysis

**Figure 7:**
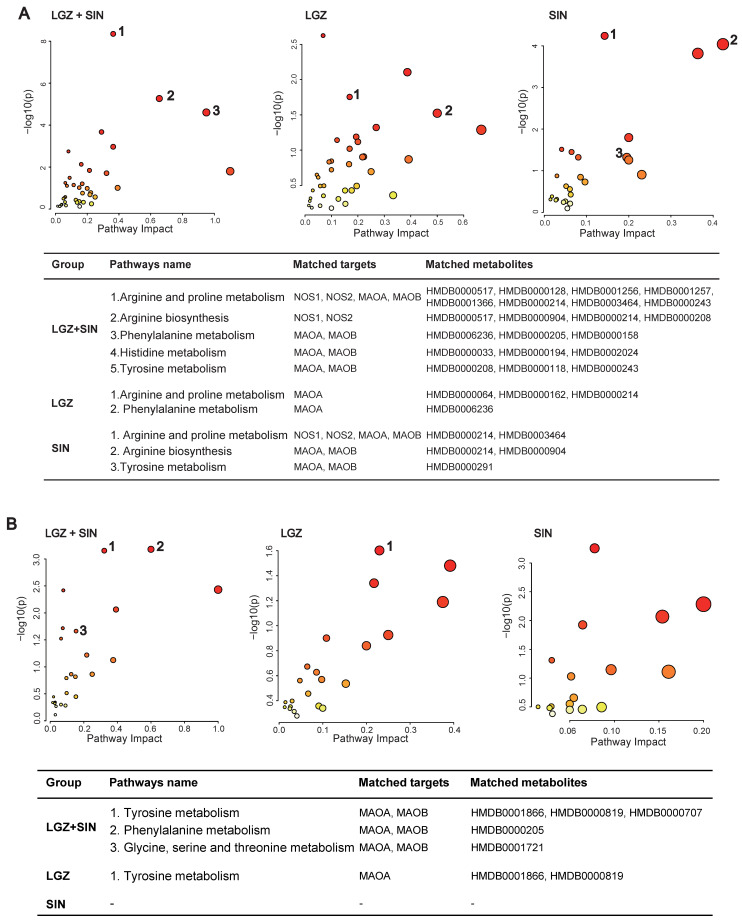
Joint pathway analysis: matched targets and metabolites. (**A**) Joint pathway analysis of the plasma samples. (**B**) Joint pathway analysis of the CSF samples. The size of the bubbles represents the number of enriched metabolites—the larger the bubble, the greater the number of enriched metabolites. The color of the bubbles indicates enrichment significance, where a darker color represents a smaller *p*-value and higher significance.

Using the “Joint-Pathway Analysis” module in MetaboAnalyst 6.0, we conducted an association analysis between the 34 core targets of LGZ and SIN, as predicted by network pharmacology, and the differentially regulated metabolites identified in plasma metabolomics following their combined administration (Figure 7A). The analysis identified 11 pathways with a *p* < 0.05, among which five pathways were enriched with both network pharmacology targets and differential metabolites from metabolomics. These pathways included arginine and proline metabolism, phenylalanine metabolism, arginine biosynthesis, histidine metabolism, and tyrosine metabolism. Further analysis of nine core targets of LGZ with the plasma differential metabolites revealed five pathways with a *p* < 0.05, of which two pathways—phenylalanine metabolism, and arginine and proline metabolism—were enriched with both targets and metabolites. For SIN, the analysis of its 25 core targets with plasma differential metabolites resulted in eight pathways with a *p* < 0.05, with three pathways simultaneously enriched with network pharmacology targets and metabolomics with differential metabolites. These included arginine and proline metabolism, arginine biosynthesis, and tyrosine metabolism.

These combined findings suggest that the phenylalanine metabolism, tyrosine metabolism, and arginine and proline metabolism pathways may be key metabolic pathways in the pain-relieving effects of LGZ and SIN. LGZ may primarily influence the tyrosine metabolism in the cerebrospinal fluid and phenylalanine metabolism in the plasma, while SIN appears to mainly regulate the tyrosine metabolism in the plasma.

The analysis of the number of pathways with a *p* < 0.05, enriched by both network pharmacology and plasma metabolomics, showed that LGZ+SIN regulated six more pathways than ligustrazine and three more than SIN. Furthermore, LGZ+SIN enriched three more pathways, with both network pharmacology targets and differential metabolites compared to LGZ and two more than SIN.

An association analysis of the 34 core targets of LGZ and SIN, as predicted by network pharmacology, with the differentially regulated metabolites in CSF metabolomics following LGZ+SIN treatment was conducted (Figure 7B). The results revealed eight pathways with a *p* < 0.05, of which three pathways—tyrosine metabolism; phenylalanine metabolism; and glycine, serine, and threonine metabolism—were significantly enriched with both network pharmacology targets and differential metabolites. These three pathways were considered the primary metabolic pathways through which LGZ+SIN regulates pain. Among these, tyrosine metabolism contained the highest number of targets and differential metabolites, indicating its pivotal role. For LGZ, the association analysis with the CSF metabolites revealed three pathways with a *p* < 0.05, with only one pathway (tyrosine metabolism) being enriched with both network pharmacology targets and differential metabolites. For SIN, the analysis showed five pathways with a *p* < 0.05, but none of these pathways were enriched with both network pharmacology targets and CSF differential metabolites.

Overall, pathway enrichment analysis demonstrated that LGZ+SIN regulated more pathways with a *p* < 0.05 than either LGZ (by five pathways) or SIN (by three pathways). Additionally, pathways enriched with both network pharmacology targets and differential metabolites were more abundant in LGZ+SIN, with three more pathways than LGZ and two more pathways than SIN.

## 3. Discussion

NP is a secondary condition associated with various clinical disorders which significantly impacts the quality of life of patients. However, the precise pathogenesis of NP remains poorly understood and involves complex interactions between multiple signaling pathways. Currently, several widely accepted mechanisms are believed to contribute to the development of NP, including inflammation, peripheral sensitization, central sensitization, dysfunction of the descending inhibitory system, and changes in ion channels. Clinical metabolomics studies have recently reported significant alterations in the plasma metabolite profiles of patients with NP, particularly in the levels of amino acid analogs such as histidine, asparagine, glutamine, tyrosine, phenylalanine, proline, and choline [20]. Furthermore, phenylalanine and tyrosine levels in the CSF of patients with localized pain syndromes have been shown to be markedly elevated [21]. Some important metabolites, such as tyrosine, purine, asparagine, histidine, serine, and glutamate, have been shown to be involved in the onset and development of neuropathic pain [22]. Therefore, exploring pathological mechanisms from the perspective of metabolomics, identifying clinical biomarkers, and further developing therapeutic drugs are highly necessary.

Multi-drug combination therapies, encompassing both combined pharmaceutical agents and multi-target TCM, can partially address clinical treatment needs. Chuanxiong Rhizoma, derived from the rhizome of *Ligusticum chuanxiong* Hort., has been widely used in traditional medicine since the Han dynasty (~1800 years ago), though it is typically employed as an adjunctive or supporting medicine according to TCM theory. Its formulations, such as ligustrazine injection and salvia miltiorrhiza ligustrazine injection, are primarily used in China for the treatment of occlusive cerebrovascular diseases [23,24]. Sinomenii Caulis, sourced from the stems of *Sinomenium acutum* (Thunb.) Rehd. et Wils., is used clinically for the treatment of rheumatism, rheumatoid arthritis, and related pain symptoms [25,26]. Chuanxiong Rhizoma and Sinomenii Caulis, widely used in clinical practice as TCM, have long been recognized for their substantial efficacy in treating various pain-related conditions. Based on these findings, we selected their active components, LGZ and SIN, for combined application, to explore their potential therapeutic effects on NP. These natural products, or TCM, frequently exhibit multi-target properties, complicating the precise identification of their therapeutic effects [27]. However, for drugs with unclear mechanisms of action, identifying their key therapeutic targets is essential.

In previous studies, we investigated the analgesic effects of the combined use of LGZ and SIN in models of inflammatory pain, sciatic nerve injury, and spinal cord injury NP [14]. Given that the CCI model simulated both neuropathic and inflammatory pain characteristics, we examined the analgesic effects of LGZ and SIN, both in combination and individually, in the CCI model rats to comprehensively assess the benefits of their combined use. In previous research, LGZ and SIN have been administered via intraperitoneal injection [12], even though both have established oral administration protocols [28,29]. Therefore, in this study, we evaluated the analgesic effects of the oral administration of LGZ and SIN in combination. Additionally, the experimental design evaluated the analgesic effects of different time points (0, 0.5, 2, 4, and 6 h), dosages (LGZ 25 mg⋅kg^−1^⋅d^−1^ + SIN 25 mg⋅kg^−1^⋅d^−1^; LGZ 50 mg⋅kg^−1^⋅d^−1^ + SIN 50 mg⋅kg^−1^⋅d^−1^; and LGZ 100 mg⋅kg^−1^⋅d^−1^ + SIN 100 mg⋅kg^−1^⋅d^−1^) and days (1, 2, and 3 days) for both combined and single-drug treatments to comprehensively characterize the analgesic properties of the LGZ-SIN combination. Results from the MWT test, cold allodynia test, and incapacitance test demonstrated that both the combined and individual treatments of LGZ and SIN effectively alleviated mechanical allodynia, cold pain sensitivity, and spontaneous pain in CCI-induced NP. Furthermore, the combination of LGZ and SIN exhibited significant greater analgesic effects than single-drug treatments, reinforcing the rationale for their combined use. We also found a dose dependence of the combination of LGZ and SIN in the MWT test. Moreover, the results suggested that the combined use of LGZ and SIN also had beneficial effects on plasma inflammation, sciatic nerve inflammation and repair in CCI rats. Clinical studies have shown that IL-6 levels are elevated in the plasma of NP patients, which is consistent with the trend observed in our CCI rat model, where IL-6 levels were detected in both the sciatic nerve and plasma. However, we did not observe a significant change in IL-6 levels, which may be attributed to the relatively short treatment duration, which may not have allowed sufficient time for the drug’s regulatory effects to fully manifest [30].

Following the evaluation of LGZ, SIN, and their combination, we conducted network pharmacology and metabolomics studies to explore their potential mechanisms in treating NP. The network pharmacology approach elucidated the analgesic mechanism of LGZ and SIN by examining their individual contributions. First, both LGZ and SIN demonstrated multi-target properties. Second, pathway analysis confirmed that both LGZ and SIN could regulate multiple signaling pathways to exert their synergistic effects. Based on network pharmacology results, modulation of the tyrosine metabolism and phenylalanine metabolism pathways may be the key mechanisms through which the combined use of LGZ and SIN exerted its analgesic effects. In addition, the combination of LGZ and SIN regulate arginine and proline metabolism, as well as histidine metabolism.

As NP affects both the peripheral and central systems, this study analyzed plasma and CSF samples to investigate the metabolic regulatory effects of LGZ and SIN, both in combination and individually, on CCI rats. The results of metabolic pathway analysis showed that the combined treatment of LGZ and SIN regulated more metabolic pathways in both CSF and plasma samples compared to either LGZ or SIN used alone, exhibiting a synergistic effect. Finally, joint pathway analysis revealed that tyrosine metabolism and phenylalanine metabolism were the key pathways enriched in both CSF and plasma samples. These pathways were considered the most critical. Among these, LGZ had a greater impact on tyrosine metabolism in CSF, while SIN exhibited a stronger effect on the tyrosine metabolism in plasma. The arginine and proline metabolism pathways contained the most targets and differential metabolites enriched by the combined treatment of LGZ and SIN in plasma samples. Therefore, the combined treatment of LGZ and SIN may alleviate pain in CCI model rats by co-regulating tyrosine metabolism and phenylalanine metabolism in both the CSF and plasma, as well as by modulating the arginine and proline metabolism in the plasma. Moreover, the number of differential metabolites in the metabolic pathways regulated by the combination of LGZ and SIN was much higher than that of LGZ and SIN alone, and interestingly, some of the differential metabolites were not present in LGZ or SIN alone, which were new differential metabolites generated by the combination. The enhanced effect of combining the two also suggests that we may be able to achieve the same effect of LGZ and SIN alone at a lower dose when they are combined. Thus, the combination of LGZ and SIN may produce a synergistic effect.

Tyrosine is an essential amino acid, and phenylalanine serves as its precursor. Both tyrosine and phenylalanine serve as precursors for monoamine neurotransmitters, including dopamine, norepinephrine, and epinephrine. The descending monoaminergic pathways, particularly those involving norepinephrine and serotonin transmission, play a crucial role in the endogenous pain modulation system, a mechanism that is well documented in NP [31]. Studies have shown that CCI leads to a reduction in the neurotransmitters crucial for descending pain regulation pathways, such as serotonin and norepinephrine [32]. Serotonin and dopamine potentiate noradrenergic effects to inhibit neuropathic pain. Moreover, antidepressants that inhibit the reuptake of norepinephrine and serotonin have been shown to be effective in chronic neuropathic pain [5]. The metabolomics results showed that serotonin and its precursor, tryptophan, increased in the plasma of the model group, but were restored after treatment. The LGZ+SIN group exhibited a more pronounced recovery compared to the individual treatments. Arginine, a non-essential amino acid, serves as a precursor for nitric oxide, proline, and glutamate. Studies have demonstrated that arginine could increase pain sensitivity in animal models [33]. Small-scale patient studies have suggested that L-arginine might have an analgesic effect on chronic pain [34]. The central glutamatergic system plays a critical role in the onset and persistence of persistent pain, including both neuropathic and inflammatory pain [35]. Following nerve injury, the downregulation of GABA and opioid receptors in the spinal cord leads to increased glutamate release, which may contribute to the development of neuropathic pain [36]. Studies have shown that CCI-induced NP reduces the GABA levels and neuronal activity in the dorsal horn [37]. Furthermore, the glutamatergic system could exacerbate chronic neuropathic pain by activating N-methyl-D-aspartate receptors (NMDARs) [38]. Studies have demonstrated that NMDARs play a crucial role in modulating both peripheral and central sensitization in NP [39]. The metabolomics analysis revealed a decrease in glutamine levels in the model group, which was subsequently restored following treatment, potentially contributing to this effect. In addition, reduced arginine levels may lead to neurotransmitter depletion, contributing to inflammatory pain [40]. Meanwhile, histidine plays a crucial role in the inflammatory process by regulating the synthesis of histamine neurotransmitters [41]. Therefore, arginine and histidine metabolism may be closely linked to the anti-inflammatory effects of the LGZ and SIN combination. In our experiment, the improvement of inflammatory factors in the sciatic nerve and plasma of CCI rats after treatment might be related to this.

To the best of our knowledge, this is the first report on the therapeutic effects and potential mechanisms of combining LGZ and SIN for the treatment of neuropathic pain induced by CCI using metabolomics and network pharmacology approaches. The combination of two drugs, LGZ and SIN, also offers a new combination of clinical treatment options for neuropathic pain. On one hand, special attention should be given to their specific targets and related metabolic signaling pathways to uncover the molecular-level regulatory mechanisms. On the other hand, extending the treatment duration and integrating in-depth studies at the cytokine level could provide a more comprehensive assessment of their anti-inflammatory, analgesic, and other potential effects. This multi-layered research approach will contribute to a more thorough understanding of the pharmacological mechanisms of LGZ and SIN combination therapy, thereby providing a stronger scientific foundation for its clinical application.

## 4. Materials and Methods

### 4.1. Chemicals and Materials

The Easyflow independent ventilation cage was purchased from Tecniplast, Italy. The Von Frey filaments were obtained from Ugo Basile Biological Apparatus Company. The Incapacitance Meter (BIO-SWB-TOUCH-R) was purchased from Bioseb, French. The pain testing frame was made in our laboratory.

AB Sciex HPLC-MS/MS system (Framingham, MA, USA) comprised an ExionLC-20AC high-performance liquid chromatography (HPLC) system, Ion Drive^TM^ Turbo V ion source, Sciex 6500^+^ triple quadrupole mass spectrometer, Analyst 1.7 data acquisition software, and MultiQuant 3.0.3 data processing software. The Targin VX-Ⅲ multi-tube vortexer was purchased from Beijing Targin Technology Co., Ltd. (Beijing, China). The Forma 88,000 Series −86 °C ultra-low temperature freezer was obtained from Thermo Scientific (Waltham, MA, USA). The Rotanta 460R high-speed refrigerated centrifuge was acquired from Hettich (Kirchlengern, Germany). The MC-8 integrated cryogenic centrifuge concentrator was obtained from Beijing JM Technology Co., Ltd. (Beijing, China). The Synergy2 multifunctional microplate reader was purchased from Bio Tek (Winooski, VT, USA). The desktop anesthetic machine was supplied by Harvard Apparatus (Cambridge, MA, USA). The ThermoStar body temperature maintenance device was purchased from RWD Life Science Co., Ltd, (Shenzhen, China). The optical microscope (Olympus BX50) was purchased from Olympus Optical Co. (Tokyo, Japan).

Ligustrazine (ligustrazine hydrochloride, lot number: DT201803-19) and sinomenine (sinomenine hydrochloride, lot number: DT201806-22), both with a purity of ≥98%, were provided by Shanxi Datian Biotechnology Ltd. (Xi’an, China). Pregabalin (lot number: 295422) was provided by Beijing J&K Scientific Ltd. (Beijing, China). The isoflurane (lot number: 217180801) was purchased from RWD Life Science Co., Ltd.

IL-6, IL-1β, and TNF-α ELISA kits were purchased from Raybiotech (Peachtree Corners, GA, USA). The tissue lysis buffer (EL-lysis) was obtained from Raybiotech. The BCA protein assay kit was purchased from Thermo Fisher (Waltham, MA, USA) and used to calibrate the content of inflammatory factors. The ProcartaPlex^TM^ Rat Cytokine and Chemokine Panel was purchased from Thermo Fisher. The 4-0 chromic gut sutures were obtained from Shandong Boda Medical Products Co., Ltd. (Heze, China).

Mass spectrometry library kits and reference standards for glucose metabolism, amino acids, bile acids, and others were purchased from Sigma for the establishment of a widely targeted metabolomics analysis platform in our laboratory. Internal standards, including d-3 norepinephrine, d-4 dopamine, d-5 serotonin, and MSK-A2, were obtained from Cambridge Isotope Laboratories. Reference standards for metabolic pathways, including tyrosine, sodium borate, benzoyl chloride, and d-5 benzoyl chloride were purchased from Sigma. All reference and internal standards had a purity greater than 99%. LC/MS-grade acetonitrile, methanol, formic acid, and ammonium formate were obtained from Beijing Dikma Technologies Inc. (Beijing, China).

### 4.2. Animals and Treatment

Adult male Sprague Dawley rats (180–200 g, 6–7 weeks) were obtained from Beijing HFK Bioscience Co., Ltd. (Beijing, China). A total of 3 rats were housed per cage in an SPF-grade lab at a constantly maintained temperature (22 ± 2 °C) with a 12 h light/dark cycle and free access to food and water.

Following the successful establishment of the model, 42 rats were randomly assigned into 7 groups, with 6 animals per group. The groups were as follows: the model group (model, 10 mL⋅kg^−1^⋅d^−1^ saline); LGZ+SIN low-dose group (LGZ 25 mg⋅kg^−1^⋅d^−1^ + SIN 25 mg⋅kg^−1^⋅d^−1^); LGZ+SIN medium-dose group (LGZ 50 mg⋅kg^−1^⋅d^−1^ + SIN 50 mg⋅kg^−1^⋅d^−1^); LGZ+SIN high-dose group (LGZ 100 mg⋅kg^−1^⋅d^−1^ + SIN 100 mg⋅kg^−1^⋅d^−1^); Ligustrazine group (LGZ, 100 mg⋅kg^−1^⋅d^−1^); Sinomenine group (SIN, 100 mg⋅kg^−1^⋅d^−1^); and Pregabalin-positive control group (Pgb, 30 mg⋅kg^−1^⋅d^−1^). In addition, 6 healthy rats were set as the sham operation group (sham, 20 mL⋅kg^−1^⋅d^−1^ saline). All animals were orally administered their respective treatments twice daily (morning and evening) for a period of three consecutive days.

### 4.3. CCI Model Establishment

The CCI model in rats was established following the method described by Bennett [42]. After anesthetizing the rats with isoflurane, they were placed on a heating pad to maintain a body temperature of approximately 37 °C. The skin below the left femur was incised and the left sciatic nerve was exposed following blunt dissection of the surrounding tissue. The nerve was then ligated with 4–0 chromic gut sutures tied in four knots, each approximately 1 mm apart. The degree of ligation was adjusted to induce slight twitching of the calf muscles without compromising the blood supply to the nerve epineurium. In the sham group, the sciatic nerve was exposed but left unligated. The MWT test was conducted both prior to surgery and on day 7 post-surgery to evaluate the success of the model.

### 4.4. Pharmacodynamic Research

The body weight of the rats was recorded daily. Behavioral tests were conducted on days 1, 2, and 3 following drug administration. The behavioral tests included the MWT test, cold allodynia test, and incapacitance test. The MWT and cold allodynia tests were conducted at 0, 0.5, 2, 4, and 6 h after drug administration each day. The incapacitance test was conducted 4 h after drug administration each day. After the final behavioral test, samples of the affected sciatic nerve were collected for hematoxylin and eosin (H&E) staining and enzyme-linked immunosorbent assay (ELISA) analysis. Plasma and CSF samples were also collected for subsequent metabolomic analysis.

The MWT test was assessed using Von Frey filaments [43]. The rats were placed in a plastic chamber (20 cm × 20 cm × 15 cm) with a transparent acrylic lid, and they were allowed to acclimate for 30 min. Von Frey filaments, ranging from 4 g to 26 g, were used during the test. The “up-and-down” method was employed to determine the MWT value of each rat [44].

The cold allodynia test was performed by spraying 0.1 mL of acetone on the affected hind paw of the rat. The responses of the rats, including paw withdrawal and licking behavior, were observed, and these were then scored based on the degree of reaction: 0 points for no response, 1 point for mild reaction or rapid withdrawal of the hind paw, 2 points for repeated paw shaking, and 3 points for sustained or repeated lifting and licking of the hind paw [45].

The incapacitance test was conducted by placing the rats in a transparent box with an inclined platform, on which the rats stood on their hind feet. The left and right hind feet were positioned on separate sensor panels. Care was taken to ensure that the rats maintained an exploratory posture without leaning against the sides of the box. A capacimeter was used to measure the weight (in grams) on each panel over a 3 s period. Each rat underwent three measurements, with a minimum of 1 min between readings. The average of the readings for each hind foot was used to calculate the weight distribution. The bipedal balance bearing value was recorded as the percentage of total body weight supported by each hind foot. In normal rats, the weight distribution is nearly symmetrical (50:50%), whereas pain resulting from injury leads to a reduction in the load-bearing capacity of the injured hind foot. The incapacitance test result was calculated using the following formula: result = weight on the affected hind foot/(weight on the left hind foot + weight on the right hind foot) × 100% [46].

The sciatic nerve tissue was fixed in 4% paraformaldehyde and subsequently embedded in paraffin to prepare 5 μm thick sections. The sections were stained with hematoxylin for 5 min, followed by eosin for 3 min. Changes in the sciatic nerve were observed under an optical microscope.

The concentrations of IL-1β, IL-6, and TNF-α in the sciatic nerve were measured by ELISA. The experimental procedure was strictly followed according to the instructions provided with the kits. The assay of 22 cytokines and chemokines in plasma was performed and analyzed independently by Laizee Biotech (Shanghai, China) via a Luminex200 instrument and ProcartaPlex Analyst 1.0 software.

### 4.5. Network Pharmacology Analysis

First, the potential targets of LGZ and SIN were identified using the SWISS Target Prediction database (http://swisstargetprediction.ch/, accessed on 11 April 2024). These targets were then verified and refined using the UniProt database to obtain accurate target names for each compound. Subsequently, pain-related target information was retrieved from the Genecards database (https://www.genecards.org/, accessed on 11 April 2024) and the OMIM database (https://omim.org/, accessed on 15 April 2024). After removing duplicates, the remaining targets were considered pain-related targets for further analysis. The intersection of LGZ and SIN alkaloid targets with those associated with pain was identified using the Bioinformatics platform (http://www.bioinformatics.com.cn, accessed on 15 April 2024), yielding common genes across the different compounds. This gene set was then analyzed based on the uniqueness of the drug–target interactions.

Next, the intersecting target genes were entered into the STRING database to construct a protein–protein interaction (PPI) network. The network was visualized using Cytoscape 3.8.2, and the CytoHubba plugin was used to identify the core targets for further differential analysis.

Finally, the core target genes underwent Gene Ontology (GO) and Kyoto Encyclopedia of Genes and Genomes (KEGG) enrichment analysis using the Metascape database. A significance threshold of *p* < 0.01 was established for all analyses. The GO analysis covered three subcategories: biological process (BP), molecular function (MF), and cellular component (CC). Furthermore, based on the relationships between protein targets and signaling pathways, a compound–target–pathway association network was built.

### 4.6. Plasma and CSF Metabolomics Analysis

#### 4.6.1. Plasma and CSF Sample Preparation

For sample preparation, 50 μL of the test sample was mixed with 450 μL of ice-cold extraction solvent containing internal standards (methanol: acetonitrile: water = 2:2:1). The mixture was vortexed for 3 min and then placed at −20 °C for 2 h. The samples were then centrifuged at 20,000× *g* for 15 min at 4 °C. The supernatant was carefully transferred to a 1.5 mL Eppendorf tube and subjected to vacuum concentration at 35 °C and 1500 rpm for 2 h. The residue was reconstituted with 100 μL of extraction solvent that was devoid of internal standards. The sample was centrifuged again at 20,000× *g* for 15 min at 4 °C, and 80 μL of the supernatant was collected for analysis. Additionally, 10 μL of each sample was pipetted to pool a quality control (QC) sample.

#### 4.6.2. Widely Targeted Metabolomics Analysis

The metabolites were identified using an in-house reference database. An ACQUITY UPLC BEH Amide column (2.1 × 50 mm, 1.7 μm, Waters, Milford, MA, USA) and a pre-column (2.1 mm × 5 mm, 1.7 μm, Waters, USA) were used for sample separation. The mobile phase consisted of Solvent A (95% water: 5% acetonitrile, with 5 mM of ammonium formate and 0.01% formic acid) and Solvent B (95% acetonitrile: 5% water, containing 5 mM of ammonium formate and 0.01% formic acid). The gradient elution program was as follows: 0–2 min, 95–95% B; 2–4 min, 95–90% B; 4–6 min, 90–90% B; 6–9 min, 90–85% B; 9–12 min, 85–85% B; 12–15 min, 85–75% B; 15–16 min, 75–75% B; 16–18 min, 75–50% B; 18–20 min, 50–50% B; 20–22 min, 50–25% B; 22–24 min, 25–25% B; 24–25 min, 25–95% B; and 25–30 min, 95–95% B. Flow rate: 0.3 mL/min; column temperature: 35 °C; temperature: 4 °C; and injection volume: 5 μL.

Electrospray ionization (ESI) was used as the ionization source. The curtain gas (N_2_) was set to 40 psi, the collision gas (N_2_) to 9 psi, and the spray voltage was set at +5500 V and −4500 V for positive and negative ion modes, respectively. The nebulizer temperature was set to 550 °C, with ion source gas (Ion Source Gas1, N_2_) and auxiliary gas (Ion Source Gas2, N_2_) both maintained at 55 psi. Scanning was performed in both positive and negative ion modes. Optimized ion pairs and mass spectrometry parameters were applied for each metabolite.

#### 4.6.3. Targeted Metabolomics Analysis

The method for measuring the tyrosine pathway was adapted from previously published protocols [47], with the necessary modifications outlined below.

Sample preparation: a 50 μL aliquot of the sample was mixed with 150 μL of acetonitrile (1:3, *v*/*v*). The mixture was vortexed at 8000 rpm for 5 min, followed by centrifugation at 20,000× *g* for 10 min. Subsequently, 10 μL of the supernatant was transferred and added to 10 μL of 100 mM sodium borate and 10 μL of 1% benzoyl chloride. The mixture was vortexed for 5 min, incubated at 25 °C for 5 min, and then centrifuged at 20,000× *g* for 10 min. The resulting supernatant (24 μL) was mixed with 6 μL of an internal standard solution (a mixture of tyrosine pathway standards and d-5 benzoyl chloride for derivatization). The mixture was then vortexed and prepared for injection.

A PFP C18 column (2.1 × 50 mm, 1.8 μm, Waters, Milford, MA, USA) was used to separate the samples. Water with 0.1% formic acid and 5 mM of ammonium formate served as Mobile Phase A and acetonitrile served as Mobile Phase B. The gradient programs were as follows: 0–1 min, 20–20% B; 1–2 min, 20–50% B; 2–6 min, 50–70% B; 6–6.5 min, 70–95% B; 8–8.1 min, 95–20% B; and 8.1–10 min, 20–20% B. Flow rate: 0.3 mL/min; column temperature: 35 °C; sample temperature: 4 °C; and injection volume: 2 μL.

Electrospray ionization (ESI) was used as the ionization source. The curtain gas (N_2_) was set to 35 psi, collision gas (N_2_) to 9 psi, and the spray voltage was set at 5500. The nebulizer temperature was set to 550 °C, with ion source gas (Ion Source Gas1, N_2_) and auxiliary gas (Ion Source Gas2, N_2_) both at 55 psi. The analysis was performed in multiple reaction monitoring (MRM) mode with positive ion scanning. The specific ion pair parameters used for the analysis are provided in Supplementary Table S1.

#### 4.6.4. Metabolomics Analysis

To ensure QC for the metabolomics analysis, a QC sample was injected after every ten experimental sample during the chromatography run. All LC-MS data were meticulous preprocessing using MultiQuant 3.0.3 software, including key steps such as peak detection, peak identification, peak area calculation, and calibration.

PCA was initially performed to identify the major variability patterns within the dataset. OPLS-DA was then applied to identify metabolites that might differentiate between groups. The quality of the OPLS-DA model was evaluated using the parameters R^2^Y and Q^2^. Additionally, permutation testing was conducted to assess the risk of false positives in the OPLS-DA model. Potential biomarkers with significant statistical and biological relevance were selected based on the following criteria: VIP > 1, *t*-test (*p*) < 0.05, and fold change (FC) > 1.2 or <0.83. Finally, metabolic pathways associated with the differentially expressed metabolites were determined using a significance threshold of *p* < 0.05. Metabolomics data were analyzed using the Metware Metabolomics Cloud Platform (https://cloud.metware.cn/, accessed on 20 December 2024) and MetaboAnalyst 6.0 (https://www.metaboanalyst.ca/, accessed on 27 December 2024).

### 4.7. Joint Pathway Analysis

A joint pathway analysis was performed using the “Joint-Pathway Analysis” module in MetaboAnalyst 6.0, in order to correlate the key targets predicted by network pharmacology with the differential metabolites identified in the metabolomics analysis. Pathways exhibiting the highest enrichment of both targets and metabolites were considered key pathways.

### 4.8. Statistical Analysis

Statistical analyses were performed using SPSS 20.0 and GraphPad Prism 8.0. All data are presented as the mean ± standard error of the mean (SEM). The significance analysis of differences between two groups was assessed using a *t*-test, while multiple comparison was conducted using one-way or two-way analysis of variance (ANOVA). A *p* value < 0.05 indicated statistical significance, and *p* < 0.01 indicated highly statistical significance.

## Data Availability

The original contributions presented in this study are included in the article/Supplementary Material. Further inquiries can be directed to the corresponding author.

## Supplementary Materials

The following supporting information can be downloaded at: https://www.mdpi.com/article/10.3390/ijms26062604/s1.

## References

[1] Finnerup N.B., Kuner R., Jensen T.S. (2021). Neuropathic Pain: From Mechanisms to Treatment. Physiol. Rev..

[2] Alles S.R.A., Smith P.A. (2018). Etiology and Pharmacology of Neuropathic Pain. Pharmacol. Rev..

[3] Orhurhu M.S., Chu R., Claus L., Roberts J., Salisu B., Urits I., Orhurhu E., Viswanath O., Kaye A.D., Kaye A.J. (2020). Neuropathic Pain and Sickle Cell Disease: A Review of Pharmacologic Management. Curr. Pain Headache Rep..

[4] Hao S., Lin S., Tao W., Zhuo M. (2025). Cortical Potentiation in Chronic Neuropathic Pain and the Future Treatment. Pharmaceuticals.

[5] Baron R., Binder A., Wasner G. (2010). Neuropathic pain: Diagnosis, pathophysiological mechanisms, and treatment. Lancet Neurol..

[6] Jiang B.-C., Liu T., Gao Y.-J. (2020). Chemokines in chronic pain: Cellular and molecular mechanisms and therapeutic potential. Pharmacol. Ther..

[7] Yoshimoto Y., Okai H., Namba H., Taguchi K., Yamauchi Y., Wakita J., Okazaki R. (2024). Combined antiallodynic effects of Neurotropin^®^–tramadol and Neurotropin^®^–mirogabalin in rats with L5-spinal nerve ligation. J. Pharmacol. Sci..

[8] Hung Y.C., Kuthati Y., Zhang X., Gao T. (2022). Analgesic Alkaloids Derived From Traditional Chinese Medicine in Pain Management. Front. Pharmacol..

[9] Xing Z., Chen Y., Chen J., Peng C., Peng F., Li D. (2024). Metabolomics integrated with mass spectrometry imaging reveals novel action of tetramethylpyrazine in migraine. Food Chem..

[10] Rao Y. (2023). Tetramethylpyrazine and Astragaloside IV have synergistic effects against spinal cord injury-induced neuropathic pain via the OIP5-AS1/miR-34a/Sirt1/NF-κB axis. Int. Immunopharmacol..

[11] Gao T., Hao J., Wiesenfeld-Hallin Z., Wang D.-Q., Xu X.-J. (2013). Analgesic effect of sinomenine in rodents after inflammation and nerve injury. Eur. J. Pharmacol..

[12] Gao T., Li T., Jiang W., Fan W., Xu X.-J., Zhao X., Yin Z., Guo H., Wang L., Gao J. (2021). Antinociceptive Effects of Sinomenine Combined With Ligustrazine or Paracetamol in Animal Models of Incisional and Inflammatory Pain. Front. Physiol..

[13] Chen J., Guo P., Liu X., Liao H., Chen K., Wang Y., Qin J., Yang F. (2023). Sinomenine alleviates diabetic peripheral neuropathic pain through inhibition of the inositol-requiring enzyme 1 alpha–X-box binding protein 1 pathway by downregulating prostaglandin-endoperoxide synthase 2. J. Diabetes Investig..

[14] Gao T., Shi T., Wiesenfeld-Hallin Z., Li T., Jiang J.-D., Xu X.-J. (2019). Sinomenine facilitates the efficacy of gabapentin or ligustrazine hydrochloride in animal models of neuropathic pain. Eur. J. Pharmacol..

[15] Meng X., Ma J., Kang S.Y., Jung H.W., Park Y.-K. (2020). Jowiseungki decoction affects diabetic nephropathy in mice through renal injury inhibition as evidenced by network pharmacology and gut microbiota analyses. Chin. Med..

[16] Hu S., Zuo H., Qi J., Hu Y., Yu B. (2019). Analysis of Effect of Schisandra in the Treatment of Myocardial Infarction Based on Three-Mode Gene Ontology Network. Front. Pharmacol..

[17] Zhang P., Zhang D., Zhou W., Wang L., Wang B., Zhang T., Li S. (2024). Network pharmacology: Towards the artificial intelligence-based precision traditional Chinese medicine. Brief. Bioinform..

[18] Yan X.-Y., Xiang P., Yu Z.-G., Yan H. (2022). Application of Metabonomics in Substance Abuse Toxicology Research. J. Forensic. Sci..

[19] Lei C., Chen Z., Fan L., Xue Z., Chen J., Wang X., Huang Z., Men Y., Yu M., Liu Y. (2022). Integrating Metabolomics and Network Analysis for Exploring the Mechanism Underlying the Antidepressant Activity of Paeoniflorin in Rats With CUMS-Induced Depression. Front. Pharmacol..

[20] Chen P., Wang C., Ren Y., Ye Z., Jiang C., Wu Z. (2021). Alterations in the gut microbiota and metabolite profiles in the context of neuropathic pain. Mol. Brain.

[21] Meissner A., Van Der Plas A.A., Van Dasselaar N.T., Deelder A.M., Van Hilten J.J., Mayboroda O.A. (2014). 1H-NMR metabolic profiling of cerebrospinal fluid in patients with complex regional pain syndrome–related dystonia. Pain.

[22] Ghafouri B., Thordeman K., Hadjikani R., Bay Nord A., Gerdle B., Bäckryd E. (2022). An investigation of metabolome in blood in patients with chronic peripheral, posttraumatic/postsurgical neuropathic pain. Sci. Rep..

[23] Shao H., He X., Zhang L., Du S., Yi X., Cui X., Liu X., Huang S., Tong R. (2021). Efficacy of Ligustrazine Injection as Adjunctive Therapy in Treating Acute Cerebral Infarction: A Systematic Review and Meta-Analysis. Front. Pharmacol..

[24] Ma Z., Zhang H., Zhao F., Li K., Dong N., Sang W. (2024). Safety and effectiveness of Salvia miltiorrhiza and ligustrazine injection for acute cerebral infarction in Chinese population: A PRISMA-compliant meta-analysis. Front. Pharmacol..

[25] Huang Z., Mao X., Chen J., He J., Shi S., Gui M., Gao H., Hong Z. (2022). Sinomenine hydrochloride injection for knee osteoarthritis: A protocol for systematic review and meta-analysis. Medicine.

[26] Li J.-M., Yao Y.-D., Luo J.-F., Liu J.-X., Lu L.-L., Liu Z.-Q., Dong Y., Xie Y., Zhou H. (2023). Pharmacological mechanisms of sinomenine in anti-inflammatory immunity and osteoprotection in rheumatoid arthritis: A systematic review. Phytomedicine.

[27] Gan X., Shu Z., Wang X., Yan D., Li J., Ofaim S., Albert R., Li X., Liu B., Zhou X. (2023). Network medicine framework reveals generic herb-symptom effectiveness of traditional Chinese medicine. Sci. Adv..

[28] Zhang S., Zheng Y., Du H., Zhang W., Li H., Ou Y., Xu F., Lin J., Fu H., Ni X. (2023). The Pathophysiological Changes and Clinical Effects of Tetramethylpyrazine in ICR Mice with Fluoride-Induced Hepatopathy. Molecules.

[29] Gao T., Shi T., Wang D.-Q., Wiesenfeld-Hallin Z., Xu X.-J. (2014). Repeated sinomenine administration alleviates chronic neuropathic pain-like behaviours in rodents without producing tolerance. Scand. J. Pain.

[30] Jönsson M., Gerdle B., Ghafouri B., Bäckryd E. (2021). The inflammatory profile of cerebrospinal fluid, plasma, and saliva from patients with severe neuropathic pain and healthy controls-a pilot study. BMC Neurosci..

[31] Zhao X., Wang C., Zhang J.-F., Liu L., Liu A.-M., Ma Q., Zhou W.-H., Xu Y. (2014). Chronic curcumin treatment normalizes depression-like behaviors in mice with mononeuropathy: Involvement of supraspinal serotonergic system and GABAA receptor. Psychopharmacology.

[32] Chaplan S.R., Bach F.W., Pogrel J.W., Chung J.M., Yaksh T.L. (1994). Quantitative assessment of tactile allodynia in the rat paw. J. Neurosci. Methods.

[33] Severyanova L.A., Bobyntsev I.I., Kir’yanova N.A., Dolgintsev M.E. (2006). Effects of L-arginine on various types of pain sensitivity. Bull. Exp. Biol. Med..

[34] Harima A., Shimizu H., Takagi H. (1991). Analgesic effect of L-arginine in patients with persistent pain. Eur. Neuropsychopharmacol..

[35] Osaka H., Mukherjee P., Aisen P.S., Pasinetti G.M. (1999). Complement-derived anaphylatoxin C5a protects against glutamate-mediated neurotoxicity. J. Cell. Biochem..

[36] Kohno T., Ji R.-R., Ito N., Allchorne A.J., Befort K., Karchewski L.A., Woolf C.J. (2005). Peripheral axonal injury results in reduced μ opioid receptor pre- and post-synaptic action in the spinal cord. Pain.

[37] Moon H.C., Park Y.S. (2017). Reduced GABAergic neuronal activity in zona incerta causes neuropathic pain in a rat sciatic nerve chronic constriction injury model. J. Pain Res..

[38] Medeiros P., Negrini-Ferrari S.E., Palazzo E., Maione S., Ferreira S.H., De Freitas R.L., Coimbra N.C. (2019). N-methyl-d-aspartate Receptors in the Prelimbic Cortex are Critical for the Maintenance of Neuropathic Pain. Neurochem. Res..

[39] Liu Y.J., Li Y.L., Fang Z.H., Liao H.L., Zhang Y.Y., Lin J., Liu F., Shen J.F. (2022). NMDARs mediate peripheral and central sensitization contributing to chronic orofacial pain. Front. Cell. Neurosci..

[40] Fung T.S., Ryu K.W., Thompson C.B. (2025). Arginine: At the crossroads of nitrogen metabolism. EMBO J..

[41] Brosnan M.E., Brosnan J.T. (2020). Histidine Metabolism and Function. J. Nutr..

[42] Bennett G.J., Xie Y.-K. (1988). A peripheral mononeuropathy in rat that produces disorders of pain sensation like those seen in man. Pain.

[43] Shen Y., Ding Z., Ma S., Ding Z., Zhang Y., Zou Y., Xu F., Yang X., Schäfer M.K.E., Guo Q. (2019). SETD7 mediates spinal microgliosis and neuropathic pain in a rat model of peripheral nerve injury. Brain. Behav. Immun..

[44] Deuis J.R., Dvorakova L.S., Vetter I. (2017). Methods Used to Evaluate Pain Behaviors in Rodents. Front. Mol. Neurosci..

[45] Chen J., Joshi S.K., DiDomenico S., Perner R.J., Mikusa J.P., Gauvin D.M., Segreti J.A., Han P., Zhang X.-F., Niforatos W. (2011). Selective blockade of TRPA1 channel attenuates pathological pain without altering noxious cold sensation or body temperature regulation. Pain.

[46] Buys M.J., Alphonso C. (2014). Novel Use of Perineural Pregabalin Infusion for Analgesia in a Rat Neuropathic Pain Model. Anesth. Analg..

[47] Wong J.-M.T., Malec P.A., Mabrouk O.S., Ro J., Dus M., Kennedy R.T. (2016). Benzoyl chloride derivatization with liquid chromatography–mass spectrometry for targeted metabolomics of neurochemicals in biological samples. J. Chromatogr. A.

