# Supplementary material for: Using Integrated Network Pharmacology and Metabolomics to Reveal the Mechanisms of the Combined Intervention of Ligustrazine and Sinomenine in CCI-Induced Neuropathic Pain Rats
Source: Int J Mol Sci. 2025 Mar 13;26(6):2604. doi: 10.3390/ijms26062604 (PMC11942381; doi:10.3390/ijms26062604)
Supplement: Supplementary file 1 [file ijms-26-02604-s001.zip › ijms-3465634-supplementary.pdf]

Supplemental Materials

Table S1. Labeling patterns and MRM conditions for the targeted metabolites

Metabolites	Abbreviation	Labeling Pattern	Ionization model	Transition	Declustering potential (V)	Collision energy (eV)	Retention time (min)	Annotations
Phenylalanine	Phe	mono	$[M + H]^+$	270.2 / 105.1	40	20	3.46	Amino acid; Precursor to catecholamines
Tyrosine	Tyr	bi	$[M + H]^+$	390.2 / 105.1	23	32	4.10	Amino acid; Precursor to catecholamines
Dopamine	DA	tri	$[M + H]^+$	466.1 / 105.1	56	43	5.16	Neurotransmitter
3,4-Dihydroxyphenylalanine	L-DOPA	tri	$[M + H]^+$	510.2 / 105.1	58	32	4.52	DA precursor
3,4-Dihydroxyphenylacetic acid	DOPAC	bi	$[M + NH4]^+$	394.1 / 105.1	80	35	4.35	DA metabolite
Homovanillic acid	HVA	mono	$[M + NH4]^+$	304.1 / 105.1	60	28	3.86	DA metabolite
3-Methoxytyramine	3-MT	bi	$[M + H]^+$	376.1 / 105.1	47	23	4.57	DA metabolite
Norepinephrine	NE	tri	$[M + H]^+$	482.1 / 105.1	28	25	4.55	Neurotransmitter; DA metabolite
Epinephrine	EP	tri	$[M + H]^+$	496.1 / 105.1	60	30	4.67	Neurotransmitter; NE metabolite

3,4-Dihydroxyphenylglycol	DOPEG	bi	$[M + NH_4]^+$	396.1 / 105.1	40	25	4.25	NE Metabolite
3-Methoxy-4-hydroxyphenylglycol	MOPEG	mono	$[M + NH_4]^+$	306.1 / 105.1	40	25	4.10	NE Metabolite
3,4-Dihydroxymandelic acid	DOMA	bi	$[M + NH_4]^+$	410.1 / 105.1	40	25	4.20	Minor NE metabolite
Normetanephrine	NM	bi	$[M - H_2O + H]^+$	374.1 / 105.1	40	25	4.04	NE Metabolite
Vanillylmandelic acid	VMA	mono	$[M + NH_4]^+$	320.2 / 181.0	60	15	3.84	NE Metabolite
Serotonin	5-HT	bi	$[M + H]^+$	385.1 / 105.1	45	38	4.52	Neurotransmitter
5-Hydroxyindoleacetic acid	5-HIAA	mono	$[M + NH_4]^+$	313.1 / 146.1	70	15	3.78	5HT metabolite
5-Hydroxytryptophol	5-HTOL	mono	$[M + H]^+$	282.1 /160.1	70	15	3.86	5-HT metabolite; Marker of alcohol consumption
N-Acetylserotonin	NAS	mono	$[M + H]^+$	323.1 /264.1	70	15	3.92	5-HT metabolite; Melatonin precursor
Tryptophan	Trp	mono	$[M + H]^+$	309.2 / 105.1	100	35	3.51	5-HT precursor; Kyn precursor
Tryptamine	TrpA	mono	$[M + H]^+$	265.2 / 144.1	50	19	3.90	Trace amine; Trp metabolite

5-Hydroxytryptophan	5-HTP	bi	[M + H]+	429.2 / 105.1	80	41	4.00	Trp metabolite; 5HT precursor
Kynurenine	Kyn	bi	[M + H]+	417.2 / 122.1	80	20	3.98	Trp metabolite
Kynurenic acid	KA	mono	[M + H]+	294.2 / 105.1	80	40	4.06	Neuroprotective Kyn metabolite
Quinolinic acid	QA	non	[M + H]+	168.2 / 78.1	40	40	0.46	Kyn metabolite
Xanthurenic acid	XA	bi	[M + H]+	414.2 / 105.1	80	40	4.06	Kyn metabolite
3-Hydroxyanthranilic acid	3-HAA	bi	[M + H]+	362.2 / 240.1	80	20	4.06	Kyn metabolite
3-Hydroxykynurenine	3-HK	tri	[M + H]+	537.2 / 240.1	80	40	4.09	Neurotoxic Kyn metabolite
Tryptophanamide	Trn	bi	[M + H]+	414.1/105.1	80	40	4.00	Trp metabolite
4-Aminobenzoic acid	4-AA	mono	[M + H]+	243.2/105.1	80	40	3.45	Trp metabolite
Picolinic acid	PA	non	[M + H]+	124.1/106.1	40	20	0.45	Trp metabolite
Choline	Ch	non	[M]+	104.1 / 60.1	50	30	0.72	Precursor to ACh
Acetylcholine	ACh	non	[M]+	146.1 / 87.1	41	19	0.85	Neurotransmitter
Glutamine	Gln	bi	[M + H]+	355.1/ 105.1	30	25	3.39	Neuroactive amino acid

Glutamate	Glu	mono	$[M + H]^+$	252.1 / 105.1	30	25	3.47	Neuroactive amino acid
Histidine	His	mono	$[M + H]^+$	260.1/ 105.1	40	35	1.10	Amino acid; Hist Precursor
Histamine	Hist	mono	$[M + H]^+$	216.1 / 105.1	40	31	1.55	Neurotransmitt er
Asparagine	Asn	bi	$[M + H]^+$	341.1 / 105.1	40	23	3.28	Amino acid
Aspartate	Asp	mono	$[M + H]^+$	238.1 / 105.1	40	23	1.06	Neuroactive amino acid
4-Aminobutyric acid	GABA	mono	$[M + H]^+$	208.1 / 105.1	30	18	1.65	Neurotransmitt er
Glycine	Gly	mono	$[M + H]^+$	180.1 / 105.1	40	19	1.17	Neuroactive amino acid
Taurine	Tau	mono	$[M + H]^+$	230.1 / 105.1	40	23	0.73	Neurotransmitt er
Serine	Ser	mono	$[M + H]^+$	210.1 / 105.1	80	20	0.93	Neuroactive amino acid
Kyotorphin	Kyo	bi	$[M + H]^+$	546.2 / 175.1	80	40	5.00	Dipeptide; Role in pain regulation
Melatonin	Mel	non	$[M + H]^+$	233.2 / 174.1	80	40	3.17	Trp metabolite

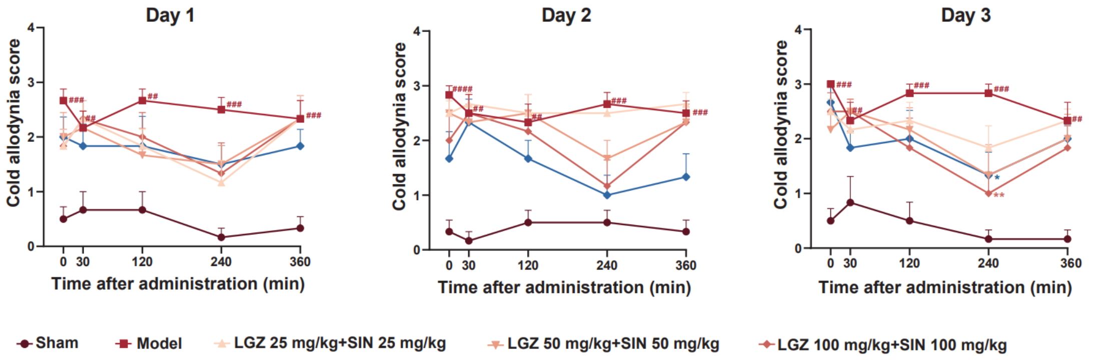

Figure S1. Cold allodynia score after administration during 3 days of treatment. $^{\#}p < 0.05$, $^{\#\#}p < 0.01$, $^{\#\#\#}p < 0.001$, and $^{\#\#\#\#}p < 0.0001$ (compared to the Sham group). $^{*}p < 0.05$, $^{**}p < 0.01$, and $^{***}p < 0.001$ (compared to the Model group).

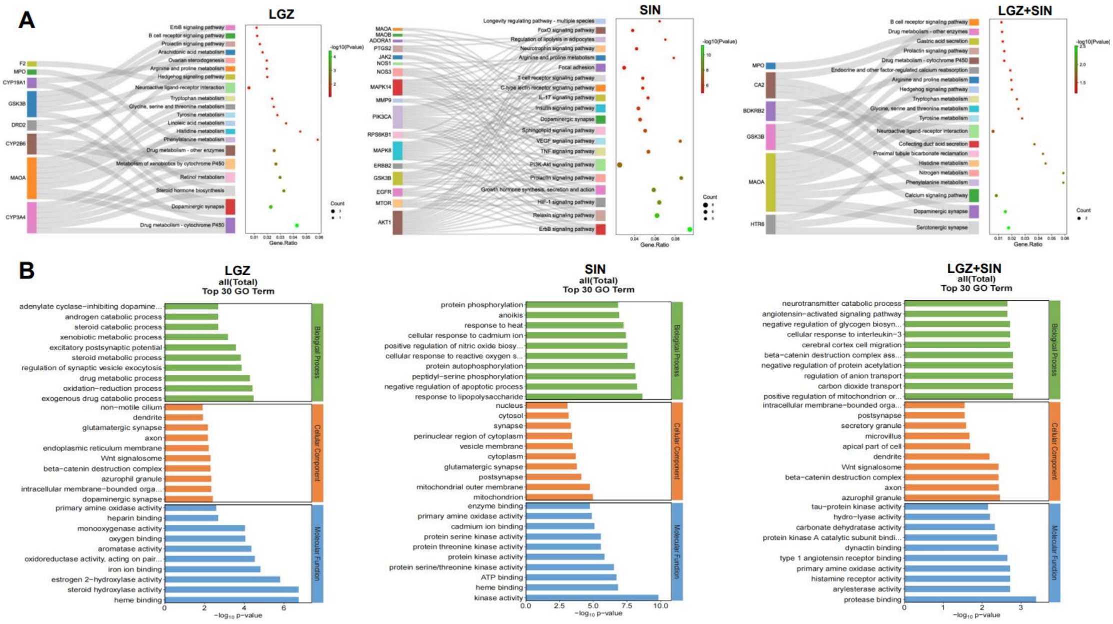

Figure S2. Exploring the effects of LGZ and SIN in pain based on network pharmacology. (A) Comparative diagram of KEGG enrichment analysis of LGZ and SIN analgesic targets. (B) Comparative diagram of GO enrichment analysis of LGZ and SIN analgesic targets.

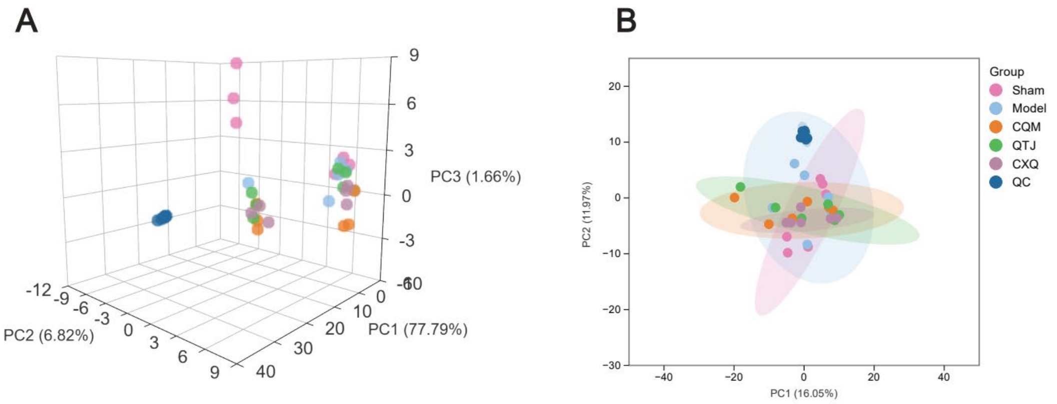

Figure S3. Principal component analysis (PCA) score plots. (A) The result of PCA in plasma. (B) The result of PCA in CSF.

Table S2. Differential metabolite changes in CSF samples

Ccompound	Model/Sham	LGZ+SIN/Model	LGZ/Model	SIN/Model
Homogentisate	↓**	↓	↓	↓
2-Aminophenol	↓*	↑**	↑**	↑*
1-Hydroxy-2-Naphthoate	↓*	↑	↑	↓
D-Pinitol	↓*	↑	↓	↑
Pterin	↑*	↓	↓	↓
Indoxyl Sulfate	↓*	↑	↑	↑
1-Methyladenosine	↓*	↑	↑	↑
1,2-Didecanoyl-sn-glycero-3-phosphocholine	↑*	↑	↓	↓
Nicotinamide Hypoxanthine Dinucleotide	↑*	↓	↓	↓
Guanosine Diphosphate	↓*	↑	↑	↑
N,N-Dimethyl-1,4-Phenylenediamine	↓	↑****	↑***	↓
Methylmalonate	↑	↑****	↑****	↑
L-Gulose	↑	↑****	↑***	↑
N-Acetylglucosamine	↑	↑*	↑	↑*
3,4-Dihydroxymandelic acid	↓	↑*	↑*	↑
Dethiobiotin	↓	↑*	↑*	↑
Normetanephrine	↓	↑*	↑***	↓
Glyceraldehyde	↑	↓*	↓	↓
Aicar	↑	↓*	↓*	↓*
Phenylpyruvate	↑	↓*	↓	↑
Riboflavin	↑	↑*	↑**	↑
N-methyl-L-glutamic Acid	↑	↓*	↓	↓*
O-Phosphoserine	↓	↑*	↑	↑
3-(4-Hydroxyphenyl)Pyruvate	↓	↑*	↑	↑
Stachyose	↓	↑*	↑	↑

Nicotinic acid	↓	↑*	↑**	↑
Orotate	↓	↑	↑*	↓
Dimethylallylpyrophosphate	↓	↓	↓*	↓
Xanthosine-Monophosphate	↓	↓	↓*	↑
5-Hydroxytryptophol	↓	↑	↓*	↑
Glucosamine	↑	↓	↓*	↓
6-Carboxyhexanoate	↓	↑	↑*	↑*
Desmosterol	↓	↑	↑*	↓
Cellobiose	↑	↑	↑*	↑
Indole-3-Acetate	↑	↑	↑*	↓
2-Methylglutarate	↓	↑	↑*	↓
Diethyl 2-Methyl-3-Oxosuccinate	↓	↑	↑*	↓
Pregnenolone Sulfate	↓	↑	↑	↑**
Elaidate	↑	↓	↓	↑*
Inosine Triphosphate	↓	↑	↑	↑*
Dihydrobiopterin	↓	↑	↑	↑*
2-Quinolinecarboxylate	↑	↓	↓	↓*
Homocysteine	↓	↑	↑	↑*
Phenylethanolamine	↓	↓	↑	↑*
4-Quinolinecarboxylate	↑	↓	↓	↓*
Thymidine	↓	↓	↓	↓*

Table S3. Differential metabolite changes in plasma samples

Ccompound	Model/Sham	CQM/Model	CXQ/Model	QTJ/Model
Sorbitol	↓**	↑	↑	↑
Cystine	↑**	↓	↑	↓*
Homovanillic acid	↑**	↑*	↑	↑
Cis-4-Hydroxy-D-Proline	↑**	↑	↑	↓
4-Hydroxyproline	↑**	↑	↑	↓
N-Acetylphenylalanine	↑**	↓	↓	↓
Cysteine	↑**	↓	↑	↓
L-Tyrosine	↑*	↑*	↑	↑
D-Pinitol	↓*	↑	↑*	↑
3-Methoxy-4-Hydroxymandelate	↑*	↓	↓	↓*
Spermine	↓*	↑*	↑	↑
Diaminopimelate	↑*	↓	↓	↓
Uridine Diphosphate Glucuronic Acid	↓*	↑	↑**	↑
N-Acetylleucine	↑*	↑	↑	↓
Theophylline	↑*	↓	↓	↓
Glutathione Reduced	↑*	↑	↓	↓
D-Ribose 5-Phosphate	↑*	↓	↓	↑
Glyceraldehyde	↑*	↓*	↓	↑
Maltose	↑*	↑	↑	↑
3-Methyl-L-Histidine	↑*	↓	↓	↓
Inosine Triphosphate	↓*	↑	↑	↑
4-Hydroxyphenylglycine	↓*	↑	↓	↑
Cytidine	↑*	↓	↓	↓
Beta-Alanine	↑*	↓	↓	↑
3-Methoxytyrosine	↑*	↑	↑	↑
Inosine	↑*	↑	↓**	↑

Pyrrole-2-Carboxylate	↑*	↓	↓	↓
Mannose	↑*	↓	↓	↑
N-Methylaspartate	↑*	↓	↓	↓
Palmitoylcarnitine	↑*	↓	↓	↓
Norvaline	↑*	↓	↓*	↓*
Aspartate	↑*	↑	↓	↓
N,N-Dimethyl-1,4-Phenylenediamine	↑	↑****	↑*	↑
Purine	↑	↑****	↑*	↓
Leucine	↑	↑***	↑**	↑
Isoleucine	↑	↑***	↑	↑**
Valine	↑	↑***	↑*	↑**
Methylmalonate	↑	↑***	↑****	↑
Resorcinol Monoacetate	↓	↑**	↑	↑
3,4-Dihydroxybenzoate	↓	↑**	↑	↑
2,3-Dihydroxybenzoate	↓	↑**	↑	↑
Pipecolate	↑	↑**	↑	↑
3-Hydroxybutyric acid	↑	↓**	↓*	↓
Lysine	↑	↑**	↑*	↑
Hydroxyproline	↓	↑**	↑	↑
1-Aminocyclopropanecarboxylate	↑	↑**	↑**	↑
Corticosterone	↑	↓**	↓	↓*
Succinate Semialdehyde	↑	↓**	↓	↓
Theobromine	↑	↑**	↑	↓
1-Hydroxy-2-Naphthoate	↓	↑**	↑	↑
D-Ornithine	↑	↑*	↑**	↑*
Alpha-Hydroxyisobutyrate	↓	↓*	↓*	↓
Norleucine	↓	↑*	↑	↑
N6,N6,N6-Trimethyl-L-lysine	↑	↑*	↑	↑

L-Arginine	↑	↑*	↑	↑
Phenylacetaldehyde	↓	↓*	↓*	↓
Anserine	↓	↑*	↑	↑
S-Carboxymethyl-L-cysteine	↓	↑*	↑	↑
4-Imidazoleacetate	↑	↑*	↑	↑
Glycerol	↑	↓*	↓*	↓
L-Ornithine	↓	↑*	↑**	↑*
Guanidinoacetate	↑	↓*	↓	↓
Indole-3-Methyl Acetate	↓	↑*	↑	↑
5-Hydroxyindoleacetate	↓	↑*	↑*	↑
Erythritol	↑	↓*	↓*	↓
Pentanoate	↑	↓*	↓	↓
Spermidine	↑	↑*	↑	↑
Carnosine	↓	↑*	↑*	↑
Mercaptopyruvate	↑	↓*	↓*	↓*
Dimethylbenzimidazole	↑	↓*	↓	↓*
Lithocholate	↑	↓*	↓	↓*
Phenylpyruvate	↑	↓*	↓	↑
Oxoglutarate	↓	↓*	↑	↑
Pyruvate	↓	↓*	↓	↑
Ethylmalonate	↑	↓*	↑	↓
Citrulline	↓	↑*	↑*	↑*
5-Methylcytosine	↑	↑*	↑	↑
Homoserine	↑	↓*	↓	↑
5-Hydroxytryptophan	↓	↑*	↑	↑
3,5-Diiodo-L-Thyronine	↓	↓*	↑	↓
Cortexolone	↓	↓*	↓	↓*
4-Guanidinobutanoate	↓	↑*	↑	↑*
Asparagine	↓	↑	↑**	↑

Hypoxanthine	↑	↑	↓**	↑
Indoleacetaldehyde	↓	↓	↓**	↓
Benzyl Alcohol	↓	↓	↓*	↓*
5-Hydroxytryptophol	↑	↓	↓*	↓
Cortisol	↑	↓	↓*	↓
Ribitol	↓	↑	↑*	↑
Arabitol	↓	↑	↑*	↑
Proline	↓	↑	↑*	↑
Xylitol	↑	↑	↑*	↑
Glucose	↓	↑	↑*	↑
Sorbate	↑	↓	↓*	↓
4-Aminobenzoate	↓	↓	↓*	↓
Creatine	↑	↓	↓*	↓
3-Methyl-2-Oxindole	↑	↓	↓*	↓
Epinephrine	↓	↑	↑*	↑
Pantothenate	↓	↑	↑*	↑
L-Alanine	↑	↑	↑*	↑
Adenosine 5'-Diphosphate	↓	↑	↑*	↑
Glycolaldehyde Dimer	↑	↓	↓	↓**
dCDP	↑	↑	↑	↑*
Deoxycarnitine	↑	↓	↑	↓*
Thiamine Monophosphate	↓	↑	↑	↑*
Threitol	↑	↓	↓	↓*
3-Hydroxybenzoate	↑	↓	↓	↑*
Xanthine	↓	↑	↑	↑*

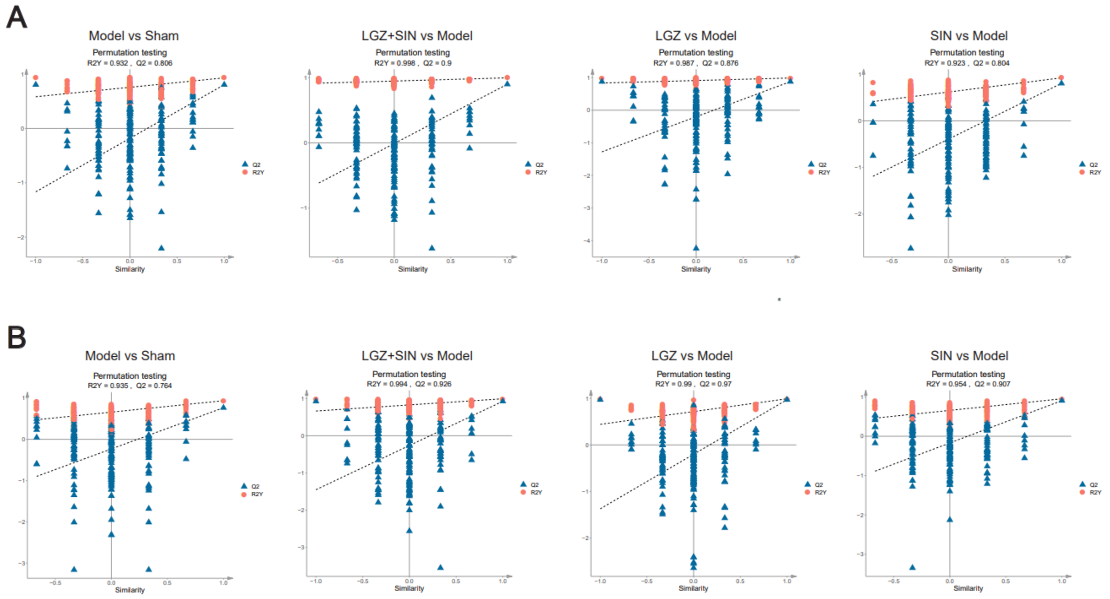

Figure S4. Results of OPLS-DA permutation test (n = 200). (A) The results of OPLS-DA in plasma. (B) The results of OPLS-DA in CSF.

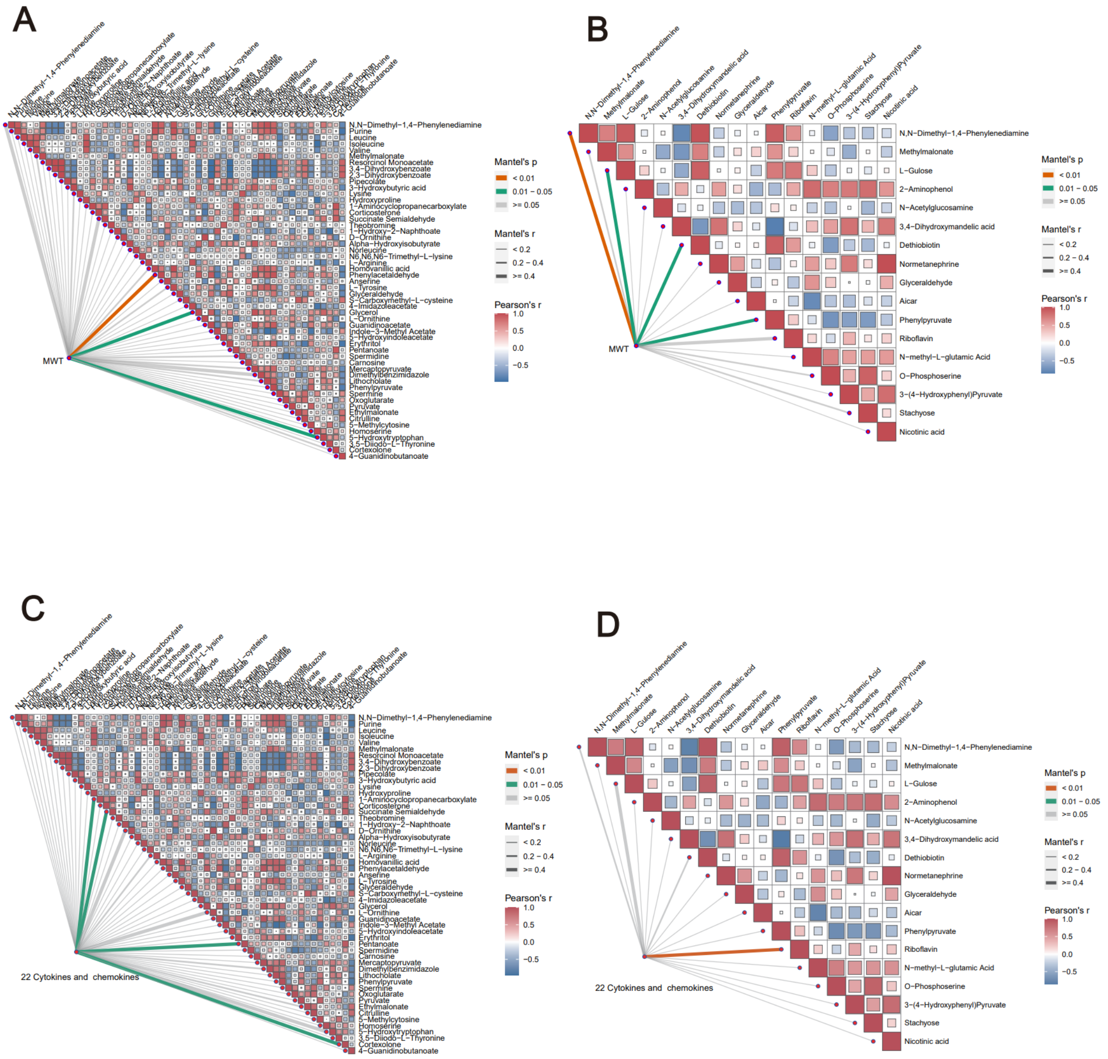

Figure S5. Correlation analysis of differential metabolites. The triangles are heat maps of correlations between substances, with red comparing positive correlations and blue indicating negative correlations. The color of the connecting line in the lower left corner indicates the result of the significance test of the correlation coefficient. (A) Correlation analysis of differential metabolites in plasma with MWT values. (B) Correlation analysis of differential metabolites in CSF with MWT values. (C) Correlation analysis of differential metabolites in plasma with 22 cytokines and chemokines values. (D) Correlation analysis of differential metabolites in CSF with 22 cytokines and chemokines values.
